# Experimental paradigms revisited: oxidative stress-induced tRNA fragmentation does not correlate with stress granule formation but is associated with delayed cell death

**DOI:** 10.1093/nar/gkac495

**Published:** 2022-06-14

**Authors:** Nasim Sanadgol, Lisa König, Aleksej Drino, Michaela Jovic, Matthias R Schaefer

**Affiliations:** Medical University of Vienna, Center for Anatomy and Cell Biology, Division of Cell and Developmental Biology, Schwarzspanierstraße 17, A-1090 Vienna, Austria; Medical University of Vienna, Center for Anatomy and Cell Biology, Division of Cell and Developmental Biology, Schwarzspanierstraße 17, A-1090 Vienna, Austria; Medical University of Vienna, Center for Anatomy and Cell Biology, Division of Cell and Developmental Biology, Schwarzspanierstraße 17, A-1090 Vienna, Austria; Medical University of Vienna, Center for Anatomy and Cell Biology, Division of Cell and Developmental Biology, Schwarzspanierstraße 17, A-1090 Vienna, Austria; Medical University of Vienna, Center for Anatomy and Cell Biology, Division of Cell and Developmental Biology, Schwarzspanierstraße 17, A-1090 Vienna, Austria

## Abstract

tRNA fragmentation is an evolutionarily conserved molecular phenomenon. tRNA-derived small RNAs (tsRNAs) have been associated with many cellular processes, including improved survival during stress conditions. Here, we have revisited accepted experimental paradigms for modeling oxidative stress resulting in tRNA fragmentation. Various cell culture models were exposed to oxidative stressors followed by determining cell viability, the production of specific tsRNAs and stress granule formation. These experiments revealed that exposure to stress parameters commonly used to induce tRNA fragmentation negatively affected cell viability after stress removal. Quantification of specific tsRNA species in cells responding to experimental stress and in cells that were transfected with synthetic tsRNAs indicated that neither physiological nor non-physiological copy numbers of tsRNAs induced the formation of stress granules. Furthermore, the increased presence of tsRNA species in culture medium collected from stressed cells indicated that cells suffering from experimental stress exposure gave rise to stable extracellular tsRNAs. These findings suggest a need to modify current experimental stress paradigms in order to allow separating the function of tRNA fragmentation during the acute stress response from tRNA fragmentation as a consequence of ongoing cell death, which will have major implications for the current perception of the biological function of stress-induced tsRNAs.

## INTRODUCTION

The biogenesis of tRNA-derived small RNAs (tsRNAs), their biological impact and their potential as biomarkers have been the subject of intense scrutiny in recent years. An increasing body of work has assigned functional relevance to various tsRNAs because their occurrence correlated not only with cellular stress responses but also with complex molecular and cellular processes including immunity, cancer, neurodegeneration and the intergenerational inheritance of information (1,2). tsRNAs have been detected in almost every cellular context, during various developmental stages and, importantly, during exposure to defined stress conditions (3). Specifically, the production of tsRNAs in the form of tRNA halves has been reported after starvation (4), oxidative stress (5,6), nutritional deficiency (7), hypoxia and hypothermia (8,9), heat shock and gamma-irradiation (6,10,11).

Stress-induced tsRNAs are the result of tRNA hydrolysis in the anticodon loop, which is performed by members of two nuclease families (RNase A and RNase T2). Angiogenin (ANG), a member of the RNase A family, is the main nuclease of various redundant nucleases capable of tRNA hydrolysis (6,8,12). Upon stress, ANG phosphorylation causes the dissociation from its inhibitor RNH1 (13) and the activation of its catalytic activity results in targeting of pyrimidine-purine dinucleotide sequences, preferentially in the loop structures of tRNAs (6,14–16).

Of note, some stressors result in tRNA fragmentation while others do not. For instance, heat shock, oxidative stress, methionine or nitrogen starvation, and stationary phase conditions caused detectable tRNA fragmentation in yeast, while glucose or general amino acid starvation as well as UV exposure did not (5). A particular stressor causing robust tRNA fragmentation in eukaryotic cells is inorganic sodium arsenite, NaAsO_2_ (As[III]). All arsenic compounds elicit a number of responses including growth inhibition, induction of DNA strand breaks and the production of reactive oxygen species, indicating toxicity, which can result in cell death (17,18). On the molecular level, As[III] stress-induced signaling cascades cause transient formation of stress granules (SG), inhibition of protein translation and, importantly, the production of tsRNAs (3). Notably, published data reporting on As[III]-induced tRNA fragmentation exposed cells to As[III] concentrations of at least 0.5 mM (6,19–34), which had been deemed non-lethal by an earlier study (35). Moreover, almost no report complemented this kind of transient treatment with additional analyses beyond two hours of the stress recovery.

To systematically assess the effects of As[III] on cellular physiology, we determined the response of various cell lines to transient As[III] exposure. In addition, we revisited the exposure of cells to hydrogen peroxide (H_2_O_2_), which serves as a common oxidative stress paradigm, and also induces tRNA fragmentation (5,25,36). Our data indicate that both As[III] as well as H_2_O_2_, if applied at concentrations that induce tRNA fragmentation, resulted in extensive cell death after removal of the respective insult. Notably, time-limited exposure to these stressors, even at lower concentrations, resulted in increased tsRNA levels after stress removal, likely due to ongoing cell death. Cell death was associated with the appearance of tsRNAs in the culture medium confirming that these small RNAs remain stable outside of cells. Taken together, these findings add important detail to the biogenesis of stress-induced tsRNAs, which might guide future experiments that aim at deciphering the biological impact of tsRNAs.

## MATERIALS AND METHODS

### Reagents

Sodium (meta)arsenite (NaAsO_2_) and sodium arsenic (V) oxide (As_2_O_5_ were purchased from Sigma-Aldrich (S7400 and 483257, respectively). For arsenite (As[III]) use, a 1 M stock solution was prepared in water and stored at RT. For arsenic (V) oxide (As[V]) use, a 218 mM stock solution was prepared in water and stored at RT. Staurosporine was purchased from Sigma (S5921), dissolved in DMSO and stored at –20°C as a 1 mM stock solution. RNAse A was purchased from Thermo Scientific™ (EN0531). RNAse V1 was purchased from Thermo Scientific™ (AM2275) and stored at –20°C in storage buffer (10 mM Tris Succinate, pH 7.5, 0.2 M KCl and 50% glycerol, v/v). The following antibodies were used: anti-TIA-1 (Abcam, ab40693, rabbit, 1:500), anti-G3BP1 (Abcam, ab56574, mouse, 1:1000), anti-cleaved Caspase-3 (Asp175) (Cell Signaling, 5A1E, rabbit, 1:500), anti-γ-H2AX(p-S139) (Upstate BioTech JBW301, mouse, 1:1000). Of note, ab40693 has been discontinued by Abcam after internal validation on TIA-1 knock-out cells (contact Abcam for further information).

### Cell culture

Human embryonic kidney (HEK293T), human cervix adenocarcinoma (HeLa) and osteosarcoma (U-2OS) cell lines were obtained from the American Type Culture Collection (ATCC) (Manassas, VA, USA) and cultured in a humidified 5% CO_2_ incubator at 37°C in standard Dulbecco's modified Eagle's medium (DMEM) supplemented with antibiotic/antimycotics, 2 mM l-glutamine and 10% fetal bovine serum (all Gibco™) denominated as DMEM-a. The establishment of immortalized mouse embryonic fibroblasts (i-MEF) was described in (83). i-MEF were cultured in a humidified 5% CO_2_ incubator at 37°C either in DMEM-a or in DMEM-b: standard DMEM supplemented with antibiotic/antimycotics, 2 mM l-glutamine, 10% fetal bovine serum and 1× non-essential amino acids (Gibco™). Primary mouse embryonic fibroblasts (p-MEF) were obtained from 9.5 to 13.5 days old embryos using standard procedures as described in (84) and analysed in passages 1 and 2. Primary mouse lung fibroblasts (p-MLF) were obtained by rinsing adult mouse lung alveola with Collagen solution and the subsequent collection of flushed-out cells as described in (85). p-MLF were cultured in DMEM supplemented with antibiotic/antimycotics, 2 mM l-glutamine and 20% fetal bovine serum (all Gibco™) and analyzed at passage 2. For passaging, cells were treated with 0.05% trypsin-EDTA in 1× PBS (both Gibco™).

### Stress experiments

#### Exposure to inorganic arsenite (As[III])

Cells were plated one day in advance to reach 70–80% confluency at the time of the experiment. Fresh medium containing increasing concentrations (0.05–0.75 mM) of sodium metaarsenite was added to cells for the indicated time periods.

#### Exposure to inorganic arsenic (V) oxide (As[V])

Cells were prepared as for other stress experiments. Fresh medium containing increasing concentrations (0.05–2.0 mM) of arsenic (V) oxide was added to cells for the indicated time periods.

#### Exposure to ratios of As[III]-to- As[V]

Cells were prepared as for other stress experiments. Fresh medium containing As[III] and As[V] mixed in different ratios but to a final concentration of 0.5 mM was added to cells for the indicated time periods.

#### Exposure to hydrogen peroxide (H_2_O_2_)

Cells were plated one day in advance to reach 70–80% confluency at the time of the experiment. Cells were exposed to 0.2 or 5 mM H_2_O_2_ in fresh medium for the indicated time periods.

#### Starvation paradigm

Complete medium was removed from cells at 70–80% confluency, followed by two brief washes in 1× Hanks Balanced Salt Solution (HBSS, Gibco™), and incubation in 1× HBSS for the indicated time periods.

#### Staurosporine treatment

Cells were incubated in fresh medium containing staurosporine (1 μM final) at 70–80% confluency for the indicated time periods.

Cells that were continuously exposed to these stress paradigms were subsequently analyzed for acute stress responses.

For analysis of stress recovery, acutely stressed cells were briefly washed in 1× PBS or 1× HBSS, respectively and cultured in fresh culture medium under standard conditions for the indicated recovery periods.

### Determining ATP levels after As[III] exposure

Cells were seeded at 3000 cells per well in 96-well black/clear bottom plates (with TC surface, Thermo Scientific™). After 24 h, cells were treated for 1 h with different concentrations of As[III], followed by washout of As[III] and re-plating in fresh medium. After another 24 h, cells were processed for determining cell viability using CellTiter-Glo^®^ 2.0 cell viability assay (Promega). ATP luminescence was measured on an EnSpire Multimode Plate Reader (PerkinElmer^®^).

### Cell viability assay

#### Trypan blue staining

Cultured cells were detached by trypsinization at indicated time points and 0.1 ml aliquots were incubated with 0.1 ml of 0.4% (w/v) Trypan blue (Invitrogen, 0.2% final). Cells were counted using an automated cell counter (Invitrogen) in at least three technical replicates.

#### Cytotoxicity assay

Cells were seeded at 3000 cells per well in 96-well black/clear bottom plates (with TC surface, Thermo Scientific™). After 24 h, cells were treated for 1 h with different concentrations of arsenic compounds (As[III], As[V]), or their combination, followed by washout and culturing cells in fresh medium. After another 24 h, cells were processed for determining release of cytotoxic protease activity using CytoTox-Glo™ cytotoxicity assay (Promega) according to the manufacturers’ recommendations. Luminescence intensity was measured using an EnSpire Multimode Plate Reader (PerkinElmer^®^).

### Small RNA enrichment

5–10 μg of total RNA from HeLa cells were subjected to small RNA isolation using SPRI beads (Beckman Coulter) according to the manufacturer's instructions.

### RNA transfection

Synthetic tsRNAs and control small RNA were purchased from Biomers. All transfections were performed using Lipofectamine™ 3000 Transfection Reagent (Thermo Fisher Scientific).

#### Synthetic RNA transfection

For transfection followed by RNA extraction, HeLa cells were grown to 70% confluency in six-well dishes. Different masses of synthetic small RNAs (1.5, 15, 150, 1500 ng) were transfected per well using 3.75 μl Lipofectamine and 2 μl of P3000 per μg synthetic RNA.

For transfection followed by confocal microscopy analysis, HeLa cells were grown to 70% confluency on GelTrex™-coated coverslips in 12-well dishes. Different masses of synthetic small RNAs (0.5, 5, 50, 500 ng) were transfected per well using 1.25 μl Lipofectamine and 2 μl of P3000 per μg synthetic RNA according to the manufacturer's instructions.

#### Small RNA transfection

For transfection of small RNAs (<200 nt) extracted from HeLa cells exposed to As[III] (0.5 mM) followed by confocal microscopy analysis, HeLa cells were grown to 70% confluency on GelTrex™-coated coverslips in 12-well dishes. Small RNAs (250 or 1000 ng) were either transfected after extraction, precipitation and resuspension in water, or melted in water at 80°C for 3 min, followed by addition of re-folding buffer (100 mM NaCl, 5 mM MgCl_2_) and ramping down to room temperature. 1.25 μl Lipofectamine and 2 μl of P3000 per μg RNA was added according to the manufacturer's instructions.

#### dsRNA (poly-IC) transfection

For transfection of poly-IC-containing dsRNA (∅ 2000 nt) followed by confocal microscopy analysis, HeLa cells were grown to 70% confluency on GelTrex™-coated coverslips in 12-well dishes. poly-IC dsRNA (50 ng) were transfected with 1.25 μl Lipofectamine and 2 μl of P3000 per μg RNA according to the manufacturer's instructions.

### Cell culture medium collection

Cells were grown to 70% confluency, exposed to As[III] (0.75 mM) for 1 h, washed briefly and incubated in fresh medium (HeLa and U2OS in DMEM-a, i-MEF in DMEM-b) for 24 h. Total RNA was isolated from 0.1 to 0.4 ml of cell culture medium using Trizol.

### Annexin-V and PI staining

Cells were seeded at 3000 cells per well in 96-well black/clear bottom plates (with TC surface, Thermo Scientific™). After 24 h, cells were treated for 1 h with different concentrations of As[III], followed by washout of As[III] and re-plating in fresh medium. After 6 h, cells were washed twice in 1× PBS, followed by processing for Annexin V-FITC and propidium iodide staining using reagents from BD Biosciences. Cells were co-stained with Hoechst 33342 (1 μg/ml). Images were taken by laser scanning microscopy using an Olympus Confocal FV3000.

### Enzymatic treatment of cell culture supernatant

Aliquots of HeLa cell culture medium (0.2 ml) were collected and centrifuged for 1 h at 16 000 × g at 4°C. As indicated, the supernatant of one aliquot was treated (either alone or in combination) with 0.2 mg/ml proteinase K, Triton X-100 (0.1% final), increasing units of RNase A (1, 10 or 50 units per 0.2 ml medium) or RNase V1 (0.5 or 1 units per 0.2 ml medium) for 30 min at 37°C. Total RNA was isolated from these aliquots of cell culture medium using Trizol and all precipitated nucleic acids were loaded onto a 12% urea–polyacrylamide gel.

### RNA extraction

#### From cells

Cells were collected in 1 ml of pre-warmed ‘home-made’ Trizol (38% phenol (v/v), 800 mM guanidine thiocyanate, 400 mM ammonium thiocyanate, 100 mM sodium acetate, 5% glycerol (v/v), 0.5% *N*-lauroylsarcosine (w/v)). Samples were incubated at room temperature for 5 min and extracted with 0.2 ml chloroform, followed by precipitation with isopropanol (overnight) and centrifugation for >20 min at 16 000 × g at 4°C. RNA pellets were washed once with 75% ethanol, re-suspended in RNase-free water (Invitrogen) and measured using NanoDrop (Thermo Scientific™).

#### From FBS or cell culture medium

For determining 5′ tsRNA levels in fetal bovine serum (FBS) aliquots (40 μl or 400 μl) were extracted using Trizol. For determining 5′ tsRNA levels in fresh or conditioned cell culture medium, aliquots were collected and centrifuged for 1 h at 16 000 × g at 4°C. 400 μl of the respective supernatant was either precipitated in the presence of sodium acetate using one volume of isopropanol followed by resuspension of the pellet in 0.1% SDS, 0.3 mM sodium acetate and RNA extraction using Trizol, or 4 × 100 μl medium were separately subjected to the RNA extraction using Trizol followed by pooling of extracted RNA.

### Northern blotting

Equal mass of purified total RNA (approximately 2 μg) were separated on 12% urea–PAA gels in 0.5% TB. RNA was transferred to nylon membranes (Roche, GE Healthcare) using semi-dry blotting with 0.5× TBE for 30 minutes at 10 V = const. Blotted RNA was immobilized by UV cross-linking (Stratalinker^®^), followed by incubation at 60°C for >1 h. Hybridization was performed over-night at 38°C with ^32^P-end labeled oligonucleotides in hybridization solution (5× SSC, 20 mM Na_2_HPO_4_ pH 7.4, 1% SDS, 1× Denhardt's reagent). After washing at 38°C with 3× SSC, 5% SDS (v/v) for 15 min and 3× SSC, 5% SDS (v/v) for 15 min, membranes were exposed at room temperature to a phospho-imaging screen and developed using an AmershamTyphoon Biomolecular Imager (GE Healthcare).

### Quantification of total RNA extractable by Trizol from mammalian cells

HeLa cells were collected by trypsinization, followed by counting using BRAND™ Bürker Counting Chambers. Total RNA was extracted in triplicates from increasing numbers of cells using Trizol. The concentration of extracted RNA was measured using NanoDrop™ (Thermo Scientific™) and QuBit™ (Invitrogen) and the sum of the total mass per cell number was calculated and plotted using Microsoft Excel.

### Semiquantitative determination of tsRNA copy numbers

Synthetic 5′ tsRNAs were serially diluted in water to prepare stock solutions of 100 pg/μl, 10 pg/μl, 1 pg/μl. The respective volumes to prepare a solution containing different masses of tsRNAs (750, 500, 250, 100, 50, 25, 10, 5, 1 pg) were mixed with 2× RNA loading dye and separated together with different masses (0.375, 0.75, 1.5 μg) of total RNA extracted from HeLa cells that were either exposed to As[III] (0.5 mM) for 1 h, or transfected with 5′ tsRNA-Gly^GCC^ or 5′ tsRNA-Ala^AGC^ using a 12% urea–PAGE, followed by blotting as described for northern blotting. Signals obtained by phospho-imaging were used to measure pixel densities at the level the tsRNA dilution series (ImageJ), which were used to create a standard curve with a linear function to calculate the mass and copy numbers of As[III]-induced or transfected 5′ tsRNAs per HeLa cell.

### Immunofluorescence

Cells were seeded onto poly-lysine-covered chamber slides (Lab-Tek) and treated as indicated. Prior to fixation, cells were washed once in 1× PBS and fixed for 15 min in 4% PFA/1x PBS followed by three washes in 1× PBS. Cells were permeabilized in PBS-Tx (0.1% Triton X100 in 1× PBS) for 5 min, followed by incubation in blocking solution (3% BSA, 0.1% Triton X-100, 0.05% sodium azide in 1× PBS) for >1 h. Cells were incubated in primary antibodies overnight at 4°C in a humidified chamber. Chamber slides were washed two times for 30 min in blocking solution, followed by incubation in secondary antibodies diluted in blocking solution for 2 h at room temperature. Chamber slides were washed in PBS-Tx two times for 30 min at room temperature. Nuclei were stained with Hoechst 33342 for 5 min, followed by a brief wash in 1× PBS. Chambers were removed and cells were mounted using VectaShield^®^ (Vector Laboratories). Images were taken by laser scanning microscopy using an Olympus Confocal FV3000.

### Automated counting of single-color images (ImageJ)

Images were quantified independently by two individuals who were blinded to the annotation of the image files and folders. DNA images (as proxy for cell number) to be counted were converted to grey scale (setting: Edit > Options > Conversions to “scale when converting”). Conversion to greyscale (Image > Type > 8-bit). Image > Adjust > Threshold (Cntl + Shift + T) was used to highlight all of the structures to be counted. Images were inverted using Dark background. To highlight black against white sliders were used to create binary versions of each image (Process-Binary-fill holes). Command Analyze > Analyze Particles was used to count cell number. Count settings were as follows: Size, 10 – Infinity; Pixel units, check; Circularity, 0.00 – 1.00; Show, nothing; Display results; Clear results; Summarize; Exclude on edges. Count data from the Summary window was taken as number of cell nuclei = cells (some nuclei sticking together are counted as one –values were adjusted accordingly). Stress granule numbers were determined by overlaying G3BP1 signals with automatically counted DNA images, followed by manual counting.

### Quantitative RT-PCR (qRT-PCR)

Total RNA isolated from FBS, fresh medium, or conditioned culture medium was treated with 0.066 U/μl TURBO DNase (Invitrogen) and 0.5 U/μl T4 polynucleotide kinase (NEB) respectively, followed by acidic phenol/chloroform purification and isopropanol precipitation. First-strand cDNA synthesis was carried out using some of the reagents provided by the NEBNext® *Multiplex Small RNA Library Prep Set for Illumina^®^ (Set 2)* kit. Briefly, 3′ SR adapter ligation followed by reverse transcription and enzyme heat-inactivation was performed according to the manual in an adjusted volume and the obtained cDNA was used for qRT-PCR. Briefly, each cDNA preparation was diluted 1:20 in water. To be able to quantify tsRNAs in each preparation, an aliquot of oligo-dT- primed cDNA obtained from HeLa cells (from 500 ng of total RNA) was added to each NEBNext® cDNA. Adding these cDNAs allowed normalizing the levels of tsRNAs to the levels of two house-keeping genes (ACTIN and GAPDH). cDNAs were subjected to qRT-PCR using forward primers complementary to the 5′ ends of several tRNA species and reverse primer complementary to the 3′ SR adaptor. qRT-PCR analyses were performed in 384-well plates using an in house-made qPCR mix in technical triplicates on a CFX384 Touch™ Real-Time PCR Detection System (BioRad). Quantitation was performed with software provided by BioRad (CFX Maestro™ Software).

### Primers and Oligonucleotides (5′ to 3′)

*Northern blotting probes (5′-3′):*

5′ tRNA-Gly^GCC/CCC^: AD0006: TCTACCACTGAACCACCAAT

3′ tRNA-Gly^GCC^: AD0007: TGGTGCATTGGCCGGG

5′ tRNA-Glu^CUC/UUC^: AD0008: GAATCCTAACCACTAGACCAC

5′ tRNA-Met-i^CAU^: HD0799: CACGCTTCCGCTGCGCCACTCTGC

U6 snRNA: AD0116: GAACGCTTCACGAATTTGCG

*Synthetic RNAs for transfections (5′-3′):*

5′ P-tsRNA-Ala^AGC^ 3′ FAM: MattVIE0435: GGGGGUGUAGCUCAGUGGUAGAGCGCGUGC

5′ P-tsRNA-Gly^GCC^ 3′ Atto590: MattVIE0436: GCAUGGGUGGUUCAGUGGUAGAAUUCUCGCCU

5′ Atto590-tsRNA-Gly^GCC^ 3′ cycP: AD0137: GCAUGGGUGGUUCAGUGGUAGAAUUCUCGCCU

5′ P-sRNA (control) 3′ Atto590: MattVIE0437: GCAUUCACUUGGAUAGUAAAUCCAAGCUGAA

*Primers for qRT-PCR (5′-3′):*

5′ tRNA-Gly^GCC^ (fwd): MATT_VIE0418: GCATGGGTGGTTCAGTGG

5′ tRNA-Ala^AGC^ (fwd): MATT_VIE0441: TATAGCTCAGTGGTAGAGCGC

5′ tRNA-Cys^GCA^ (fwd): MATT_VIE0441: TATAGCTCAGTGGTAGAGCAT

5′ tRNA-Glu^CUC^ (fwd): MATT_VIE0443: CCTGGTGGTCTAGTGGTTAGG

5′ tsRNA-His^GUG^ (fwd): MATT_VIE0444: TGATCGTATAGTGGTTAGTAC

5′ tsRNA-Val^AAC/CAC^ (fwd): MATT_VIE0445: TTCCGTAGTGTAGTGGTTATC

5′ tRNA-Asp^GUC^ (fwd): MATT_VIE0446: TCCTCGTTAGTATAGTGGTGAG

Reverse primer in NEB^Next^ SR RT primer: MATT_VIE0438: AGACGTGTGCTCTTCCGATCT

GAPDH (fwd): MATT_VIE0084: TGAACGGGAAGCTCACTGG

GAPDH (rev): MATT_VIE0085: TCCACCACCCTGTTGCTGTA

beta-Actin (fwd): MATT_VIE0088: AAGGCCAACCGTGAAAAGAT

beta-Actin (rev): MATT_VIE0089: GTGGTACGACCAGAGGCATAC

## RESULTS

### Stress-Induced tRNA fragmentation occurs in response to high micromolar As[III]-concentrations

**Figure 1.**
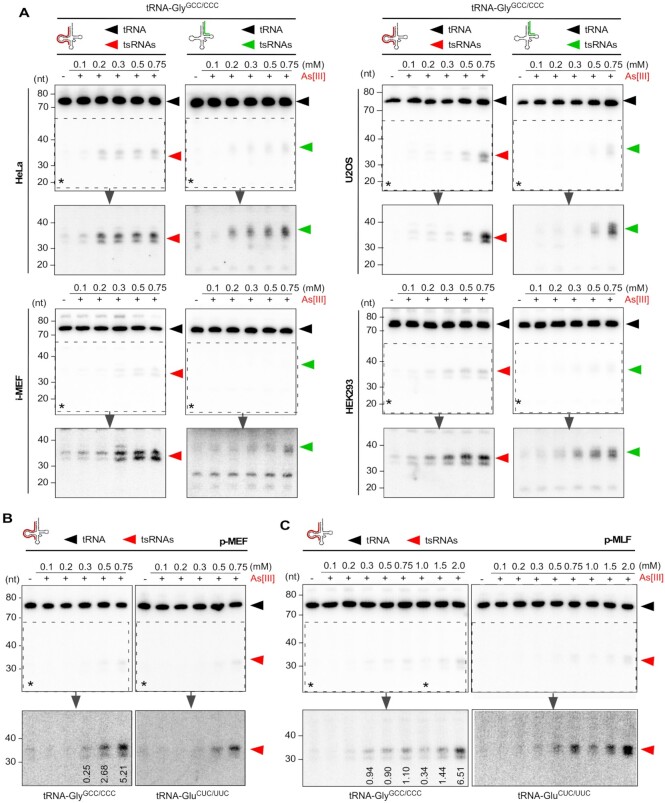
(**A**) Northern blotting of total RNA (1.5 μg) from HeLa, U2OS, HEK293 cell lines and i-MEF after exposure to increasing molarities of As[III] (0.1–0.75 mM, for 1 h) using a probe against the 5′ and 3′ ends of tRNA-Gly^GCC/CCC^. Black arrowheads: mature tRNAs; red arrowheads: 5′ tsRNAs; green arrowheads: 3′ tsRNAs; asterisks: region that was digitally enhanced in lower panels. (**B**) Northern blotting of total RNA (1.5 μg) from p-MEF after exposure to increasing molarities of As[III] (as in A) using probes against the 5′ end of tRNA-Gly^GCC/CCC^ and tRNA-Glu^CUC/UUC^, respectively. Black arrowheads: mature tRNAs; red arrowheads: 5′ tsRNAs; asterisks: region that was digitally enhanced in lower panels. Numbers in lower panels for tRNA-Gly^GCC/CCC^ indicate the percentage of tsRNAs in relation to full-length tRNAs as calculated by semiquantitative northern blotting and image analysis (related to Supplementary Figure S1B). (**C**) Northern blotting of total RNA (1.5 μg) from p-MLF after exposure to increasing molarities of As[III] (0.1–2.0 mM, for 1 h) using probes against the 5′ end of tRNA-Gly^GCC/CCC^ and tRNA-Glu^CUC/UUC^, respectively. Black arrowheads: mature tRNAs; red arrowheads: 5′ tsRNAs; asterisks: region that was digitally enhanced in lower panels. Numbers in lower panels for tRNA-Gly^GCC/CCC^ indicate the percentage of tsRNAs in relation to full-length tRNAs as calculated by semiquantitative northern blotting and image analysis (related to Supplementary Figure S1B).

Induction of tRNA fragmentation commonly involves transient exposure of cultured cells to oxidizing agents such as As[III] or H_2_O_2_ in the high micromolar concentration range (Supplementary Table S1). Specifically, reports of acute As[III] exposure resulting in tRNA fragmentation frequently employed concentrations above 0.5 mM suggesting that lower As[III] did not cause tsRNA production. We confirmed that exposure of immortalized mouse embryonic fibroblasts (i-MEF) and two cancer cell lines (HeLa and U2OS) to ≥ 0.5 mM As[III] for 2 h, as reported in (6), resulted in robust tRNA fragmentation as evidenced by northern blotting for 5′ tsRNA-Gly^GCC^, one of the most abundant stress-inducible tRNA fragments (Supplementary Figure S1A). To systematically assess which experimental parameters induce reproducible tRNA fragmentation, various immortalized cell lines were exposed to increasing As[III] concentrations for 1 h (time-limited exposure) followed by immediate RNA extraction (acute As[III] stress). The extent of tRNA fragmentation was determined by northern blotting using ^32^P-labeled probes hybridizing to either the 5′ or 3′ regions of various tRNA isoacceptors. The results showed that time-limited exposure to As[III] at concentrations ≥0.2–0.3 mM induced tRNA fragmentation, detectable by northern blotting, in all tested cell lines. Image quantification indicated that the fraction of tsRNAs derived from a given tRNA isoacceptor was depending on the cell line as exemplified by HeLa cells (remaining below 5% for 5′ tsRNAs independently of As[III] concentration), or by U2OS cells (increasing to 18% for 5′ tsRNAs in an As[III] concentration-dependent fashion (Figure 1A and Supplementary Figure S1B-C). Notably, exposing primary embryonic fibroblasts (p-MEF) or primary mouse lung fibroblasts (p-MLF) to As[III] also caused tRNA fragmentation, which was detectable by northern blotting at As[III] concentrations above 0.2–0.3 mM. However, tRNA fragmentation in primary cells did not result in 5′ tsRNA levels, which were comparable to those observed in immortalized cell lines (Figure 1B, C). While these observations confirmed tRNA fragment biogenesis in response to As[III]-induced stress in all cell types, they also indicated differences as to how immortalized and primary cells responded to As[III] exposure, specifically in terms of tsRNA levels.

### SG formation occurs at As[III] concentrations that barely induce detectable tRNA fragmentation

Stress granule (SG) formation is commonly used as a proxy for functioning cellular stress responses including those to As[III] exposure (35,37–39). Notably, previous reports also connected tRNA fragmentation to SG formation, specifically after 5′ tsRNAs were transfected into immortalized cells, which resulted in the induction of SG formation (6,19,21,40,41). The observed differences in levels of specific 5′ tsRNAs between primary and immortalized cells upon As[III] exposure prompted us to revisit the link between 5′ tsRNAs and the induction of SG. To this end, various cell lines and primary cell preparations were exposed to increasing As[III] concentrations followed by immunodetection of cytoplasmic G3BP1 and nuclear TIA-1, two proteins that re-localize to cytoplasmic SG in response to As[III] exposure (42–44). The results showed that SG were induced in immortalized cells by As[III] concentrations as low as 0.050–0.1 mM (Supplementary Figure S2A). In contrast, while primary cells also started forming SG at As[III] concentrations above 0.1–0.2 mM As[III], they did so in a heterogenous fashion (i.e. not every cell contained discernable SG) and to a lesser extent (fewer and smaller SG) (Supplementary Figure S2B, C). These observations indicated that As[III]-induced SG formation occurred at As[III] concentrations that were insufficient to cause tRNA fragmentation above steady-state background signals, at least not to an extent, which would allow detection by northern blotting. These findings also suggested that, if tsRNAs act as inducers of SG formation, they must do so in copy numbers, which are below the detection limits of northern blotting.

### Relative quantification of endogenous and As[III]-induced 5′ tsRNAs

**Figure 2.**
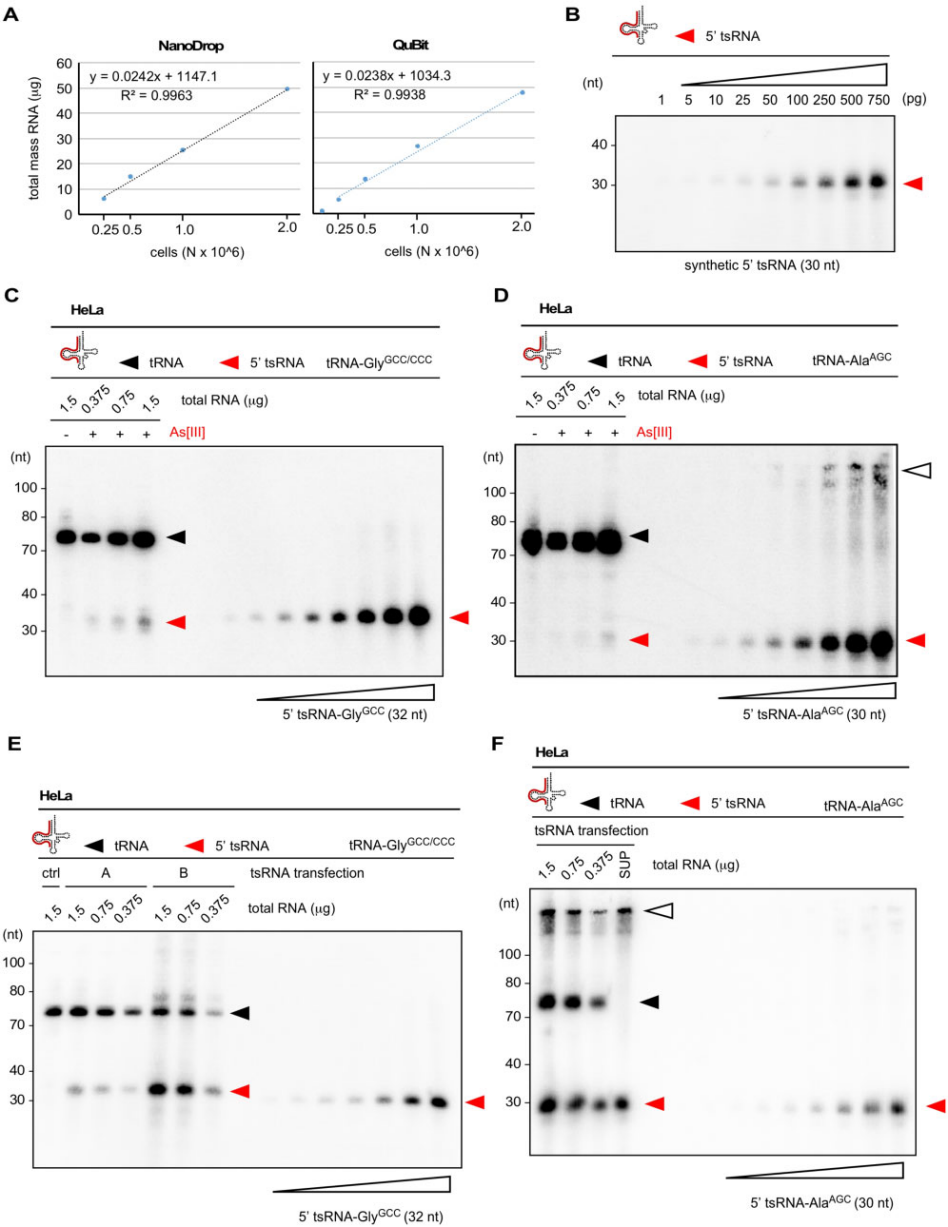
(**A**) The combined mass of total RNA extracted from defined HeLa cell numbers was measured using NanoDrop™ (left) or QuBit™ (right) and plotted against cell numbers. A function was derived allowing to calculate the mass of total RNA per HeLa cell. (**B**) Northern blotting on increasing mass (1–750 pg) of a synthetic tRNA fragment (5′ tsRNA-Ala^AGC^) using a probe against the 5′ end of tRNA-Ala^AGC^. Signals obtained by phospho-imaging were used to measure pixel densities (ImageJ), which were plotted against tsRNA mass. This allowed to create a standard curve for tsRNA signals. Red arrowheads: 5′ tsRNAs. (**C**) Northern blotting of total RNA from control HeLa cells (1.5 μg) and HeLa cells (0.375–1.5 μg) exposed to As[III] (0.5 mM, for 1 h) in combination with increasing mass (5–750 pg) of a synthetic tRNA fragment (5′ tsRNA-Gly^GCC^) using a probe against the 5′ end of tsRNA-Gly^GCC^. Signals obtained by phospho-imaging were used to measure pixel densities at the level of tsRNAs (ImageJ), which were used to create a standard curve with a linear function to calculate the mass and copy numbers of 5′ tsRNA-Gly^GCC^ per HeLa cell. Black arrowhead: mature tRNAs; red arrowheads: 5′ tsRNAs. (**D**) Northern blotting of total RNA as described in (C) in combination with increasing mass (5–750 pg) of a synthetic tRNA fragment (5′ tsRNA-Ala^AGC^) using a probe against the 5′ end of tsRNA-Ala^AGC^. Signals obtained by phospho-imaging were used to derive mass and copy numbers of 5′ tsRNA-Ala^AGC^ per HeLa cell. Black arrowhead: mature tRNAs; red arrowheads: 5′ tsRNAs; white arrowhead: higher-order structures of 5′ tsRNA-Ala^AGC^ in wells and with low mobility. (**E**) Northern blotting of total RNA extracted from control HeLa cells (1.5 μg) and HeLa cells (1.5-0.375 μg) transfected with two molarities of a synthetic 5′ tsRNA-Gly^GCC^ (A: 10 nM, B: 100 nM) in combination with increasing mass (5–750 pg) of a synthetic tRNA fragment (5′ tsRNA-Gly^GCC^) using a probe against the 5′ end of tRNA-Gly^GCC^. Signals obtained by phospho-imaging were used to measure pixel densities at the level of tsRNAs (ImageJ), which were used to create a standard curve with a linear function to calculate the mass and copy numbers of transfected 5′ tsRNA-Gly^GCC^ per HeLa cell. Black arrowhead: mature tRNAs; red arrowheads: 5′ tsRNAs. (**F**) Northern blotting of total RNA extracted from HeLa cells (1.5–0.375 μg) transfected with a synthetic 5′ tsRNA-Ala^AGC^ (10 nM), and a fraction of the medium (1/15th) that was removed at the time of analysis, in combination with increasing mass (5–750 pg) of a synthetic tRNA fragment (5′ tsRNA-Ala^AGC^) using a probe against the 5′ end of 5′ tRNA-Ala^AGC^. Signals obtained by phospho-imaging were used to measure pixel densities at the level of tsRNAs (ImageJ), which were used to create a standard curve with a linear function to calculate the mass and copy numbers of transfected 5′ tsRNA-Ala^AGC^ per HeLa cell. Black arrowhead: mature tRNAs; red arrowheads: 5′ tsRNAs; white arrowhead: higher-order structures of 5′ tsRNA-Ala^AGC^ in wells and with low mobility.

Can 5′ tsRNAs either endogenously produced or ectopically introduced into cells as reported in (6,19,40,41) induce SG formation? Notably, stress-induced tRNA fragmentation results in tsRNA levels that represent only a fraction of total parental tRNA pool (Figure 1 and Supplementary Figure S1). To address how many As[III]-induced tsRNAs are actually contained in a single cell, HeLa cells were used to approximate the total number of specific and endogenously produced 5′ tsRNAs. Plotting the total RNA mass against different numbers of HeLa cells indicated that organic solvent-based extraction yielded an average of about 25 picogram total RNA per HeLa cell (Figure 2A). Using this value in combination with northern blotting on a titration series of synthetic 5′ tsRNAs (Figure 2B) and total RNA extracted from HeLa cells that were exposed to 0.5 mM As[III] for one hour, we determined the number of two 5′ tsRNA species (derived from tRNA-Gly^GCC/CCC^ and tRNA-Ala^AGC^) per HeLa cell, which had been implicated in inducing SG formation (6,19,40,41). Using probe signals from replicate northern blotting and assuming that every cell responded equally to As[III] exposure indicated that a single HeLa cell contained about 35 000 molecules of 5′ tsRNA-Gly^GCC^ and 18 000 molecules of 5′ tsRNA-Ala^AGC^ (Supplementary Tables S2, S3, Figure 2C, D).

### SG formation can be induced by dsRNA (poly-IC) but not by synthetic tsRNAs

**Figure 3.**
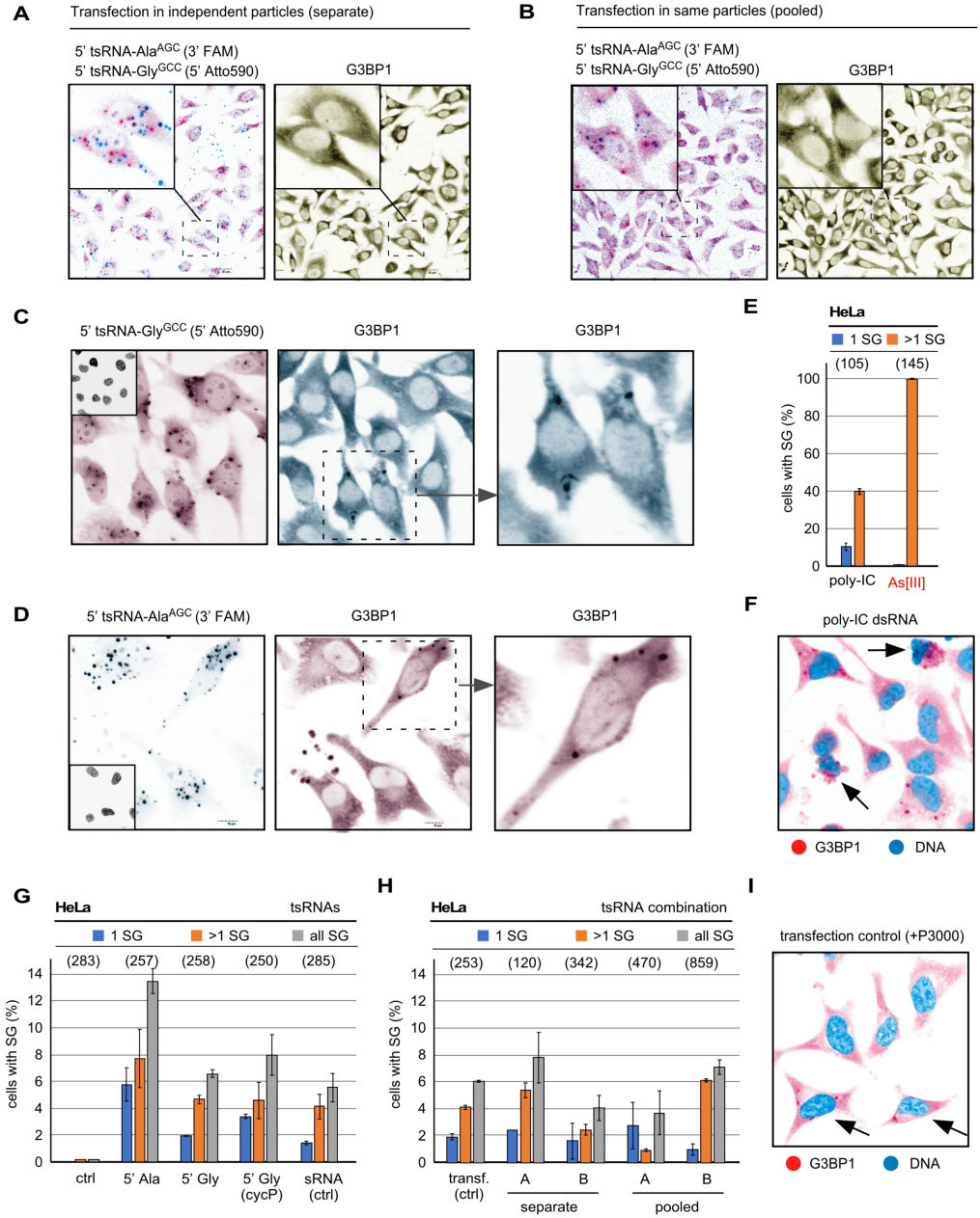
(**A**) Representative confocal image of HeLa cells after transfection with two tsRNAs (5′ tsRNA-Ala^AGC^ labeled with FAM, 5′ tsRNA-Gly^GCC^ labeled with Atto590, each 100 nM) in separately produced liposomes. Left image shows merge of both signals (5′ tsRNA-Ala^AGC^ in cyan, 5′ tsRNA-Gly^GCC^ in magenta) with inset showing separation of both 5′ tsRNA-derived signals. To monitor the induction of SG formation, indirect immunofluorescence with an antibody against G3BP1 (magenta) was used. (**B**) Representative confocal image of HeLa cells after transfection with two tsRNAs (5′ tsRNA-Ala^AGC^ labeled with FAM, 5′ tsRNA-Gly^GCC^ labeled with Atto590, each 100 nM) in the liposomes (pooled). Left image shows merge of both signals (5′ tsRNA-Ala^AGC^ in cyan, 5′ tsRNA-Gly^GCC^ in magenta) with inset showing co-localization of both 5′ tsRNA-derived signals. To monitor the induction of SG formation, indirect immunofluorescence with an antibody against G3BP1 (magenta) was used. (**C**) Representative confocal image of HeLa cells after transfection with 5′ tsRNA-Gly^GCC^ labeled with Atto590, but containing a 3′ CycP, 100 nM). Left image shows 5′ tsRNA-Gly^GCC^ in magenta, while SG formation was monitored by indirect immunofluorescence using an antibody against G3BP1 (cyan). Dashed region was digitally magnified to show the presence of G3BP1-positive SG. Inset: DNA (black). (**D**) Representative confocal image of HeLa cells after transfection with 5′ tsRNA-Ala^AGC^ labeled with FAM, 100 nM). Left image shows 5′ tsRNA-Ala^AGC^ in cyan, while SG formation was monitored by indirect immunofluorescence using an antibody against G3BP1 (magenta). Dashed region was digitally magnified to show the presence of G3BP1-positive SG. Inset: DNA (black). (**E**) Quantification of SG formed by transfection of HeLa cells with dsRNA (poly-IC,155 pM) or after time-limited exposure (1 h) to As[III] (0.3 mM). SG formation was quantified by counting cells with exactly one (1 SG) and more than one SG per cell (>1 SG) using ImageJ. Number of analyzed cells in parentheses. (**F**) Representative confocal image of HeLa cells after transfection with dsRNA (poly-IC,155 pM). SG formation was monitored by indirect immunofluorescence using an antibody against G3BP1 (magenta). Arrows point at necroptotic cells. DNA is false-colored in cyan. (**G**) Quantification of SG formed by transfection of HeLa cells with various 5′ tsRNAs or a small RNA control (related to Supplementary Figure S3A) at the highest molarity tested (100 nM) SG formation was quantified as described in (**E**). Number of analyzed cells in parentheses. (**H**) Quantification of SG formed by transfection of HeLa cells with two molarities (A: 10 nM, B: 100 nM) of tsRNAs (5′ tsRNA-Ala^AGC^ and 5′ tsRNA-Gly^GCC^) in separately produced liposomes or the same liposomes. Transf. (ctrl) designates SG counts in cells that were treated with Lipofectamine only. Number of analyzed cells in parentheses. (**I**) Representative confocal image of HeLa cells after transfection with transfection reagents only (+P3000). SG formation was monitored by indirect immunofluorescence using an antibody against G3BP1 (magenta). Arrows point at cells with SG. DNA is false-colored in cyan.

When calculating 5′ tsRNA copy numbers that were transfected into mammalian cells as published in (40,45–47), and assuming (an unlikely) 100% transfection efficiency, we arrived at 200–600 million specific 5′ tsRNA molecules per cell (Supplementary Note 1). This number exceeds the number of specific As[III]-induced 5′ tsRNAs, as determined by relative and semi-quantitative northern blotting (Supplementary Tables S2, S3, Figure 2C, D), by a factor of >10 000, and suggested that transfection of non-physiological tsRNA copy numbers might have resulted in the induction of SG. How many 5′ tsRNAs per cell are actually required to induce SG formation? To answer this question, synthetic 5′ tsRNAs, which were shown to affect SG formation and translational processes (40,45–47), as well as various RNA controls were transfected into HeLa cells and transfection efficiency as well as SG formation was determined (Supplementary Figure S3A). To approximate the number of specific 5′ tsRNAs that can be introduced into a single HeLa cell, different masses of either 5′ tsRNA-Gly^GCC^ or 5′ tsRNA-Ala^AGC^ were transfected into a defined number of cells, followed by RNA extraction and northern blotting. Using a titration series of a known mass of each of the transfected 5′ tsRNAs, the number of tsRNAs detectable by northern blotting was quantified. The results revealed that a single transfected HeLa cell harbored between 0.1 and 4 millions of each specific 5′ tsRNA species, which corresponded to about 1.5–70 femtograms per individual tsRNA identity per cell (Figure 2E, F and Supplementary Tables S4 and S5). When considering that HeLa cells were transfected with increasing tsRNA mass (1.5, 15, 150, 1500 ng) per 0.3 million cells, these values indicated that only about 1.7% of each synthetic tsRNA mass had actually been transfected into cells at the time of the analysis (7 h post-transfection). To determine the actual transfection efficiency per cell, 5′ tsRNAs and small RNA controls were transfected at various molarities representing very high to lower (likely more physiological) masses of RNAs, which were co-visualized alone or in combination with the SG marker G3BP1 using confocal microscopy. This revealed transfection of every cell as evidenced by fluorescent signals emanating from labeled small RNAs (Supplementary Figure S3B–E and Figure 3A, B). However, the formation of SG, while detectable, was independent of the amount of transfected 5′ tsRNA species since neighboring cells revealing comparable tsRNA signals showed clear differences in G3BP1 aggregation into SG (Figure 3C, D). As positive control for SG formation, double-stranded (ds) poly-IC RNA (48) and time-limited exposure to As[III] (0.3 mM) was used, whereas an unrelated fluorescently labeled small RNA, as published in (47), was transfected as negative control. Both As[III] exposure as well as dsRNA (poly-IC) transfection induced SG formation in 100% or 40% of all cells, respectively (Figure 3E). Notably, dsRNA (poly-IC) transfection also caused necroptotic cell death in many cells (Figure 3F). In contrast, the combined and quantified results of all control and small RNA transfection experiments showed that, independently of the transfected mass of specific 5′ tsRNAs (alone or in combination), SG formation was never detected in >14% of all transfected cells (Figure 3G, H). These results confirmed previous observations (40), by which only a maximum of 12% of all cells transfected with 5′ tsRNA-Ala^AGC^ or 5′ tsRNA-Gly^GCC^ showed SG. Importantly, the ability to co-detect transfected 5′ tsRNAs with G3BP1 in the same cells allowed excluding large differences in individual transfection efficiency as the reason for the induction of SG formation in some but not all cells. We also tested the effects of transfecting pools of all small RNAs (<200 nt) extracted from As[III]-exposed HeLa cells, which contained stress-induced tsRNAs into non-stressed HeLa cells (Supplementary Figure S3F). The results showed that no more than 7% of all cells contained G3BP1-positive SG, which was independent of the transfected mass of small RNAs or their structural status (Supplementary Figure S3F). Notably, we observed that small RNA control transfections (Figure 3G) as well as transfection with only Lipofectamine and the additive P3000 (Figure 3H), the latter of which needs to be increased depending on transfected RNA content, resulted in up to 8% of all cells showing SG formation above background (Figure 3I). Taken together, these results strongly suggested that specific 5′ tsRNAs do not induce SG formation in mammalian cells, even at copy numbers, which exceed the levels of endogenously produced 5′ tsRNAs by magnitudes.

### Commonly applied oxidative stress paradigms result in increased cell death

**Figure 4.**
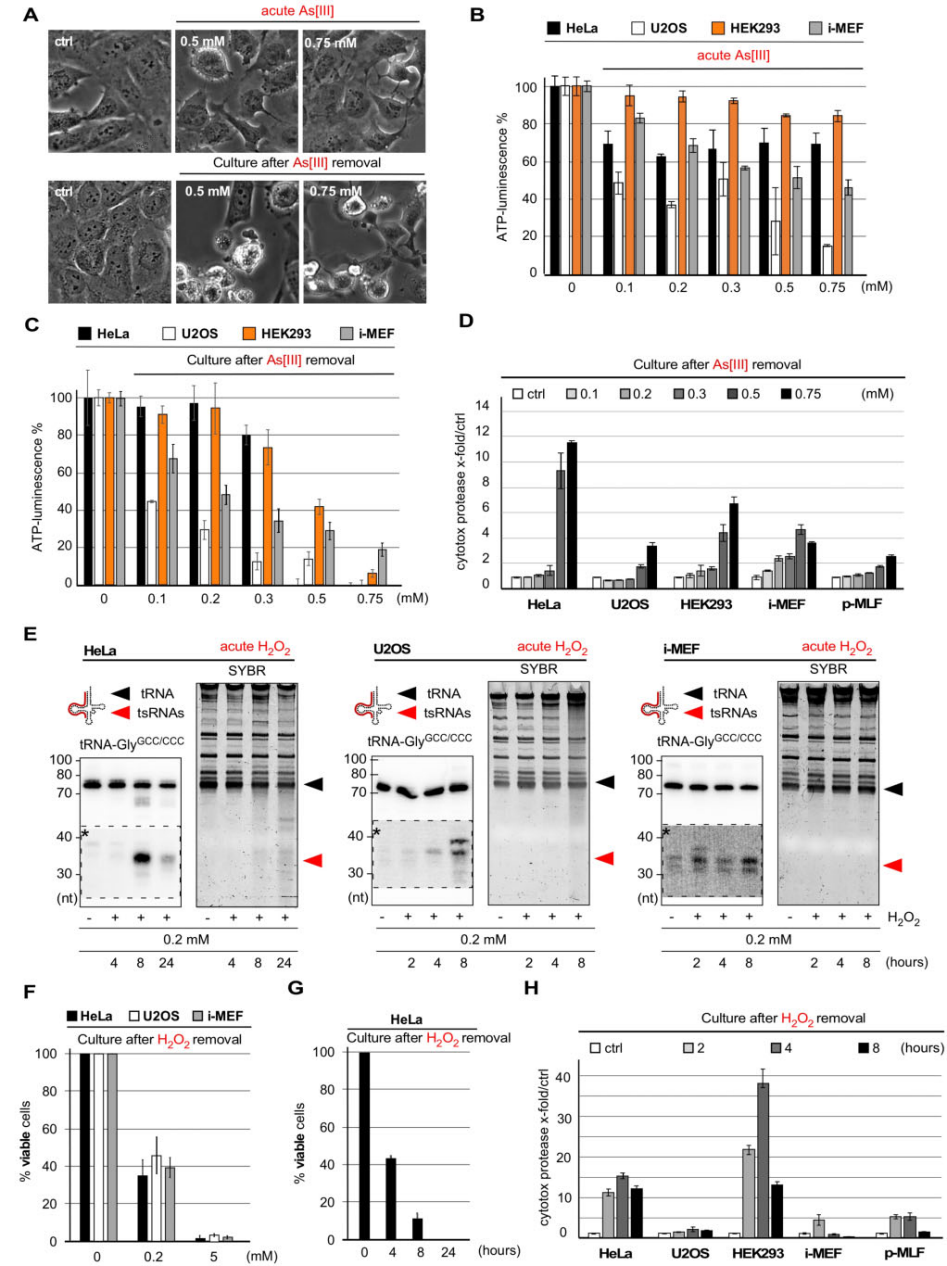
(**A**) Phase-contrast images of U2OS cells after exposure to > 0.5 mM As[III] (for 2 h) and 24 h after removal of the stressor. (**B**) Bar-chart depicting ATP measurements in HeLa, U2OS, HEK293 cells and i-MEF immediately after time-limited exposure (1 h) to increasing As[III] molarities (acute As[III]). Error bars depict standard deviation (SD) of triplicate measurements from three independent wells in the same experiment. (**C**) Bar-chart depicting ATP measurements in HeLa, U2OS, HEK293 cells and i-MEF after time-limited exposure (one hour) to As[III] (as in B) followed by culturing cells for 24 h after As[III] removal. Error bars depict standard deviation (SD) of triplicate measurements from three independent wells in the same experiment. (**D**) Bar-chart depicting the quantification of luminescence created by cytotoxic protease activity in the culture medium of HeLa, U2OS, HEK293 cells, i-MEF and p-MLF after time-limited exposure (1 h) to As[III] (as in B), followed by culturing cells for 24 h after the removal of As[III]. Error bars depict standard deviation (SD) of triplicate measurements from three independent wells in the same experiment. (**E**) Northern blotting of total RNA (1.5 μg) from HeLa, U2OS cells and i-MEF exposed to 0.2 mM H_2_O_2_ for the indicated times (2, 4, 8, 24 h) using a probe against the 5′ end of tRNA-Gly^GCC/CCC^. Individual right images: SYBR-staining of PAA gels before transfer onto membranes. Black arrowhead: mature tRNAs; red arrowhead: tsRNAs; dashed insets with asterisks: digitally enhanced against parental tRNA signals. (**F**) Bar-chart depicting cell membrane-integrity measurements by Tblue staining of HeLa, U2OS cells and i-MEF after exposure to 0.2, or 5 mM H_2_O_2_ for 4 h, followed by culturing cells for 24 h after the removal of H_2_O_2_. Error bars depict standard deviation (SD) of triplicate cell counts from three independent wells in the same experiment. (**G**) Bar-chart depicting cell membrane-integrity measurements by Tblue staining of HeLa cells after exposure to 0.2 mM H_2_O_2_ as published in (36) and for the indicated times (4, 8, 24 h), followed by culturing cells for 24 h after the removal of H_2_O_2_. Error bars depict standard deviation (SD) of triplicate cell counts from three independent wells in the same experiment. (**H**) Bar-chart depicting the quantification of luminescence created by cytotoxic protease activity in the culture medium of HeLa, U2OS, HEK293 cells, i-MEF and p-MLF after exposure (2, 4, 8 h) to H_2_O_2_ (0.2 mM), followed by culturing cells for 24 h after the removal of H_2_O_2_. Error bars depict standard deviation (SD) of triplicate measurements from three independent wells in the same experiment.

Arsenic compounds affect cellular physiology on multiple levels including inhibition of proliferation, induction of oxidative stress response pathways and damage to organelles that results even in cell death (49). Molecularly, As[III] coordinates thiol groups, which results in a rather unspecific inhibition of a range of proteins, including enzymes (50). Importantly, any phenotypic characterization of cells exposed to As[III] with regard to tRNA fragmentation has been performed during the acute stress response (Supplementary Table S1), thereby largely ignoring the potential for delayed molecular consequences resulting from As[III] exposure. To address how time-limited As[III] exposure affected cells after removal of the stressor, various immortalized cell lines were exposed to medium containing As[III] ranging from 0.1 to 0.75 mM. Metabolic activity as well as cell viability were determined immediately after As[III] removal (acute stress response) and after a recovery phase of 24 h in fresh medium. Notably, a considerable fraction of cells exposed to As[III] concentrations ≥0.5 mM displayed cell line-dependent morphology changes (rounding up, Figure 4A), often resulting in lower adherence and cell loss (HEK293 > i-MEF ≥ HeLa ≥ U2OS) at sampling time points after removal of the stressor. Especially the latter greatly interfered with analyzing As[III]-induced phenotypes in particular cell lines (i.e. HEK293), in particular when this required trypsinization (for cell counting) or fixation (for cell staining). While none of the applied As[III] concentrations affected cell viability during the acute stress response, when measured by determining general membrane integrity through Trypan blue (TBlue) staining (Supplementary Figure S4A), metabolic activity, determined by quantification of ATP levels, decreased in a cell line- and concentration-dependent fashion (Figure 4B). Negative metabolic effects were even more pronounced when cells were analyzed 24 h after removal of the stressor (Figure 4C). TBlue staining confirmed compromised membrane integrity when cells were analyzed after recovering from transient As[III] exposure at concentrations above 0.2–0.3 mM (Supplementary Figure S4B). Measuring the extracellular activity of intracellular protease activity upon release from membrane-compromised and dying cells (CytoTox-Glo™) confirmed decreased cell viability 24 h after exposure to As[III] concentrations above 0.2–0.3 mM (Figure 4D). Notably, exposing p-MLF to the highest As[III] concentrations resulted in only limited cell death when measured 24 h after stress removal and when compared to immortalized cell lines, which correlated with the absence of noticeable tRNA fragmentation during the acute stress response (Figure 1C). Furthermore, treatment of cells with ≥ 0.5 mM As[III] for 2 h, as published in (6), also resulted in impaired cell viability when measured 24 h later (Supplementary Figure S4C). Since the sampling time-point of 24 h post-exposure was characterized by major cell detachment and floating cell debris, which hampered the immunofluorescence analysis of markers for cell death, signs of apoptosis were determined 6 h after time-limited exposure to As[III] using Annexin V and propidium iodide (AxV/PI) staining. This revealed many AxV/PI double-positive cells indicating the onset of substantial cell death after removal of As[III] (Supplementary Figure S4D). Notably, exposure to high concentrations of H_2_O_2_ and experimental parameters as published in (5,25,36) caused tRNA fragmentation (Figure 4E), which correlated with a H_2_O_2_ concentration-dependent loss of cell viability when measured after removal of the stressor (Figure 4F,G and Supplementary Figure S4E, F). Cytotoxicity measurements confirmed that cells, exposed to 0.2 mM H_2_O_2_ for different time periods, displayed increased cell death when measured 24 h later (Figure 4H). Notably, cell death was cell type-dependent with both i-MEFs and p-MLF as well as the slowly proliferating U2OS cell line showing the least cell death. Lastly, incubating cells for extended time periods in Hank's balanced salt solution (HBSS), which induces oxidative stress as part of the response to nutrient starvation (51,52), resulted also in lower cell viability within 24 h after the treatment (Supplementary Figure S4G, H). These combined results indicated that commonly used stress paradigms to induce tRNA fragmentation have detrimental consequences for cellular metabolism and cell survival, specifically after removal of the respective insult and during the time which is commonly described as stress recovery period.

### Time-limited exposure to As[III] results in compromised RNA integrity

**Figure 5.**
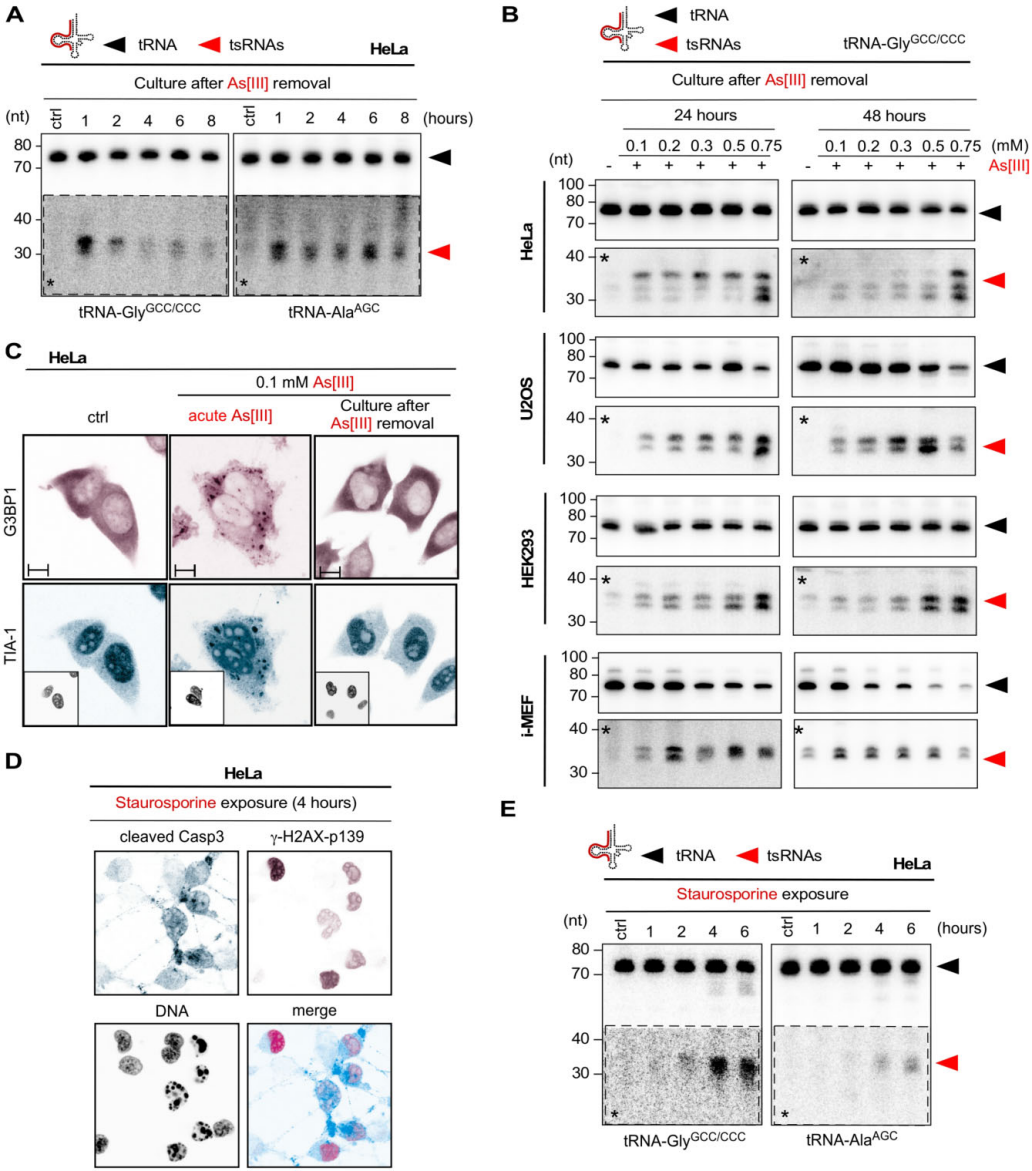
(**A**) Northern blotting of total RNA (1.5 μg) extracted from HeLa cells after time-limited exposure (1 h) to As[III] (0.5 mM), followed by culturing cells for the indicated times (1, 2, 4, 6, 8 h) after the removal of As[III], using probes against the 5′ ends of tRNA-Gly^GCC/CCC^ and tRNA-Ala^AGC^. Black arrowhead: mature tRNAs; red arrowhead: tsRNAs; asterisks: digitally enhanced against parental tRNA signals. (**B**) Northern blotting of total RNA (3 μg) extracted from HeLa, U2OS, HEK293 cells and i-MEF, which had been exposed (1 h) to increasing molarities of As[III], followed by culturing cells for 24 or 48 h after the removal of As[III] using a probe against the 5′ end of tRNA-Gly^GCC/CCC^. Black arrowhead: mature tRNAs; red arrowhead: tsRNAs; asterisks: digitally enhanced against parental tRNA signals. (**C**) Indirect immunofluorescence image of HeLa cells after time-limited exposure (1 h) to As[III] (0.5 mM), followed by culturing cells for 24 h after the removal of As[III], using antibodies against G3BP1 (magenta) and TIA-1 (cyan). Individual insets: DNA (black). Scale bar 10 μm. (**D**) Indirect immunofluorescence image of HeLa cells after exposure (4 h) to staurosporine (1 μM), using antibodies against cleaved caspase 3 (cyan) as indicator of apoptotic cells and phosphorylated γ-H2AX (magenta) as indicator of DNA damage. DNA: black. (**E**) Northern blotting of total RNA (1.5 μg) extracted from HeLa cells, which had been exposed to staurosporine (1 μM) for the indicated times (1, 2, 4, 6 h) using probes against the 5′ ends of tRNA-Gly^GCC/CCC^ and tRNA-Ala^AGC^. Black arrowhead: mature tRNAs; red arrowhead: tsRNAs; asterisks: digitally enhanced against parental tRNA signals.

Stress-induced tsRNAs from transient exposure to 0.5 mM As[III] peaked during the acute stress response and diminished in quantity within 8 h after removal of the insult (Figure 5A). Notably, an increase in tsRNA levels over controls could still be detected 24 or 48 h after removal of As[III] (Figure 5B and Supplementary Figure S5A, B). As[III]-induced SG quantitatively dissolved after about 120 min (53). The absence of SG was confirmed in HeLa cells 24 h after time-limited exposure to 0.1 mM As[III] (Figure 5C). Hence, tsRNAs that were detectable at this time after the removal of the stressor were likely the products of continuing tRNA fragmentation, which was connected to cell death. Of note, cells that had been exposed to the highest As[III] concentrations contained not only tsRNAs but also compromised RNA integrity including decreasing levels of mature tRNAs and U6 snRNA (Supplementary Figure S5C, D). To test if inducing cell death resulted in increased tRNA fragmentation, HeLa cells were exposed to staurosporine, an inducer of apoptosis. Treatment with staurosporine for > 4 h induced apoptosis as evidenced by an increase in DNA double-strand breaks and cleavage of caspase 3 (Figure 5D). Notably, cells exposed to staurosporine > 4 h showed tsRNA levels that were comparable to those induced by the cellular response to As[III] (Figure 5E). These observations suggested that time-limited exposure to As[III], even to doses that did not result in tRNA fragmentation during the acute stress response, induced long-term effects that impacted tRNA stability, which also included tRNA fragmentation, likely as result of ongoing cell death.

### Time-limited As[III] exposure results in extracellular tsRNAs

**Figure 6.**
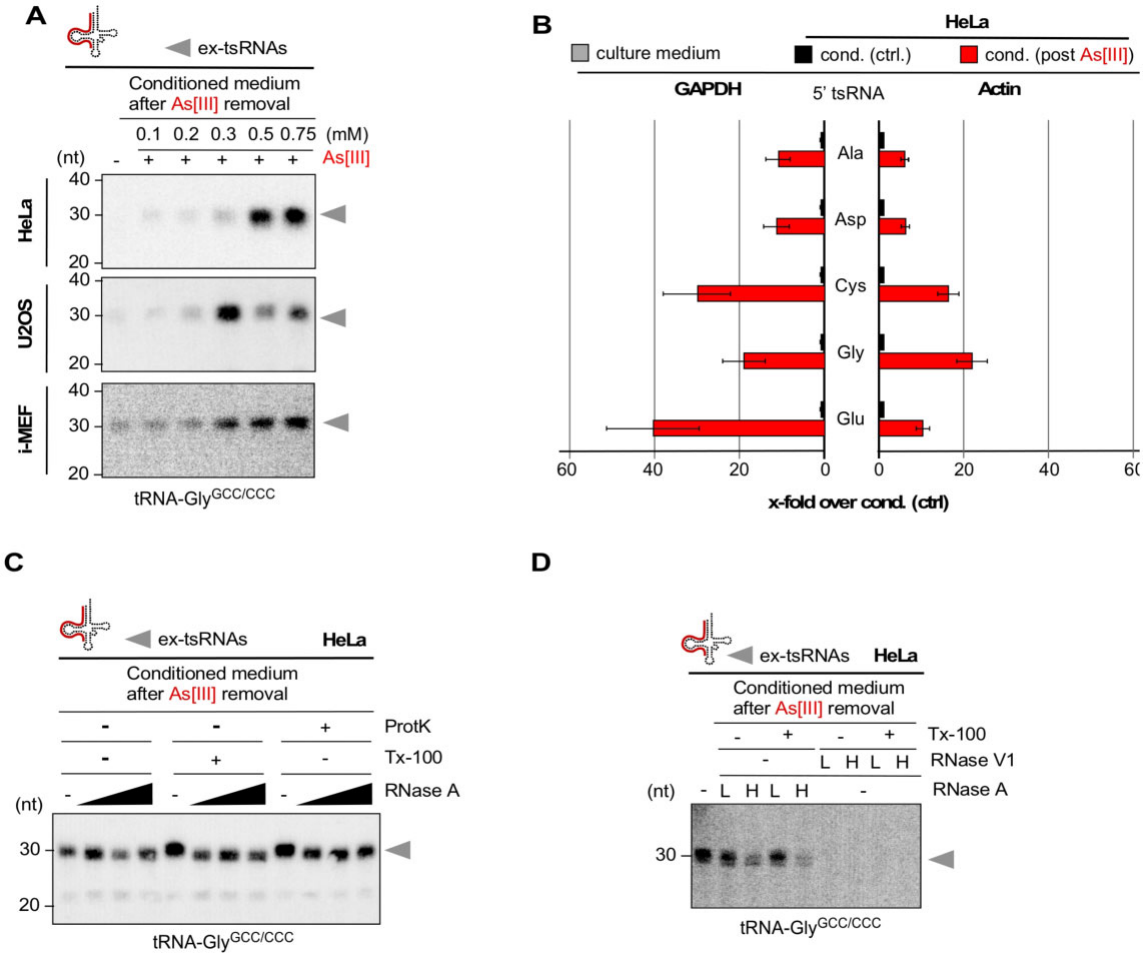
(**A**) Northern blotting of RNAs extracted from cell culture medium that was conditioned by HeLa, U2OS cells (0.2 ml medium) or i-MEF (0.4 ml medium), which had been exposed (for 1 h) to high micromolar concentrations of As[III], followed by culturing cells for 24 h after the removal of As[III] using a probe against the 5′ end of tRNA-Gly^GCC/CCC^. grey arrowheads: ex-tsRNAs. (**B**) qRT-PCR quantification of 5′ tsRNAs contained in RNAs extracted from cell culture medium (0.4 ml) that was either conditioned by HeLa cells, which had been exposed (for 1 h) to As[III] (0.75 mM), followed by culturing cells for 24 h after the removal of As[III] or by HeLa cells that were not exposed to As[III]. Bar-chart depicts the fold-change of specific 5′ tsRNAs in media (cond. post As[III]) over 5′ tsRNAs in control media (cond. ctrl), which were normalized to GAPDH (left) or ACTIN (right). Error bars depict standard error of the mean (SEM) of triplicate measurements from one experiment. (**C**) Northern blotting of RNAs extracted from HeLa cell culture medium (0.2 ml) of time-limited exposure (1 h) to As[III] (0.75 mM), followed by culturing cells for 24 h after the removal of As[III], and after being treated (either alone or in combination) with proteinase K (ProtK), detergent (Tx-100) and increasing concentrations of RNase A, using a probe against the 5′ end of tRNA-Gly^GCC/CCC^. grey arrowheads: ex-tsRNAs. (**D**) Northern blotting of RNAs extracted from HeLa cells as in (C), and after being treated (either alone or in combination) with detergent (Tx-100) and two concentrations (L, low; H, high) of either RNase A or RNase V1, using a probe against the 5′ end of tRNA-Gly^GCC/CCC^. Grey arrowheads: ex-tsRNAs.

Since tsRNAs have repeatedly been detected in extracellular compartments (30,54–58), we asked whether increased cell death by transient As[III] exposure resulted also in tsRNAs detectable in the cell culture medium (ex-tsRNAs). To test this, HeLa cells were transiently exposed to increasing As[III] concentrations, followed by 24 h of incubation after As[III] removal and the collection of cell-free culture medium (stress-conditioned medium). Northern blotting on extracted RNAs revealed the presence of specific 5′ ex-tsRNAs in medium collected from cells, which had been exposed to those As[III] concentrations that caused substantial cell death (Figure 6A). Since tRNA fragments have been detected in fetal calf serum (FBS), a pivotal component of most tissue culture media (59), quantitative reverse transcription PCR (qRT-PCR) was used to determine the amount of specific 5′ ex-tsRNAs in FBS, in unconditioned as well as in conditioned medium (normal versus stress-conditioned). The results showed that very low levels of various 5′ tsRNAs were detectable in FBS or unconditioned medium (Supplementary Figure S6A). In contrast, medium conditioned by HeLa cells that were cultured for 24 h after time-limited As[III] exposure contained many more 5′ tsRNAs than medium collected from cells grown as controls (Figure 6B). Sanger sequencing of specific 5′ tsRNA-containing PCR amplicons indicated their short sequence length confirming observations made by northern blotting (Supplementary Figure S6B). Taken together, these observations indicated that As[III]-induced cell death resulted in the release of cellular content, including tsRNAs, some of which remained stable in extracellular space.

### 5′ ex-tsRNAs are stabilized against nucleases by double-stranded RNA structures

Since 5′ ex-tsRNAs were stable for at least 24 h in the culture medium, we addressed whether they were contained in membranous vesicles or protected by proteins. Conditioned medium from HeLa cells, which were cultured after the removal of As[III] was exposed to RNase A in the presence or absence of detergent (Triton X-100), or Proteinase K, followed by RNA extraction and northern blotting. The results showed that increasing concentrations of RNase A were unable to digest 5′ ex-tsRNA-Gly^GCC/CCC^ or 5′ ex-tsRNA-Glu^CUC/UUC^ contained in culture medium, which was independent of the pre-treatment with detergent or Proteinase K suggesting that ex-tsRNAs were neither part of membranous vesicles nor RNPs (Figure 6C). To determine whether ex-tsRNAs were protected from RNase A digest by forming dsRNA structures, cell culture medium was exposed to RNase A or dsRNase V1 in the presence or absence of detergent. The results showed that dsRNase V1 digested 5′ ex-tsRNA-Gly^GCC/CCC^ completely indicating that As[III]-induced 5′ ex-tsRNAs were stabilized against RNaseA digestion by double-stranded structures (Figure 6D).

### Arsenite but not its oxidized form arsenate causes SG, tRNA fragmentation and cell death

**Figure 7.**
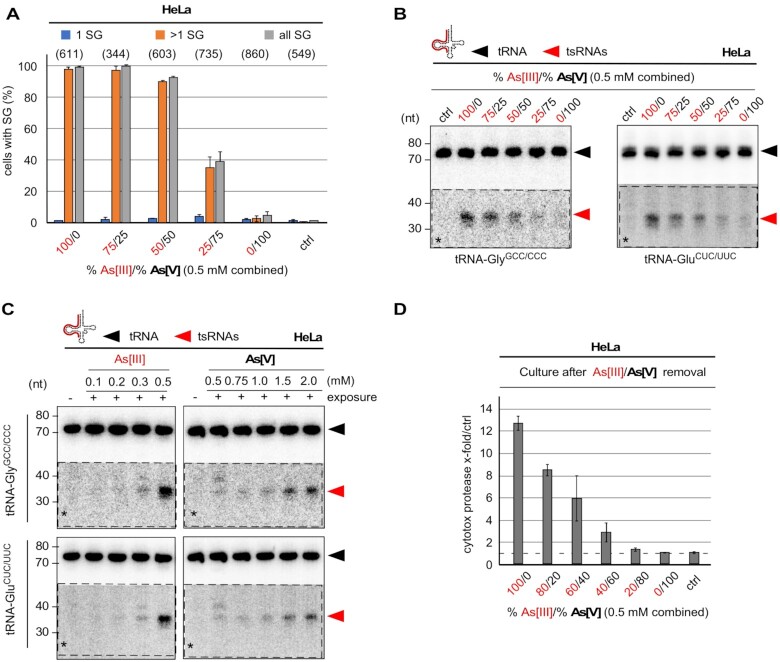
(**A**) Quantification of SG formed by time-limited exposure (1 h) of HeLa cells to different ratios of As[III] and As[V] at a combined molarity of 0.5 mM. Number of analyzed cells in parentheses. (**B**) Northern blotting of total RNA (1.5 μg) extracted HeLa cells after exposure to different ratios of As[III] and As[V] at a combined molarity of 0.5 mM using a probe against the 5′ end of tRNA-Gly^GCC/CCC^, followed by stripping and probing for the 5' end of tRNA-Glu^CUC/UUC^. Black arrowheads: mature tRNAs; red arrowheads: 5′ tsRNAs; dashed insets with asterisks: digitally enhanced against parental tRNA signals. (**C**) Northern blotting of total RNA (1.5 μg) extracted HeLa cells after exposure to increasing molarities of As[III] (left) or As[V] (right) using a probe against the 5′ end of tRNA-Gly^GCC/CCC^, followed by stripping and probing for the 5' end of tRNA-Glu^CUC/UUC^. Black arrowheads: mature tRNAs; red arrowheads: 5′ tsRNAs; dashed insets with asterisks: digitally enhanced against parental tRNA signals. (**D**) Bar-chart depicting the quantification of luminescence created by cytotoxic protease activity in the culture medium of HeLa cells after time-limited exposure (1 h) to different ratios of As[III] and As[V] at a combined molarity of 0.5 mM, followed by culturing cells for 24 h after the removal of the arsenic compounds. Error bars depict standard deviation (SD) of triplicate measurements from three independent wells in the same experiment.

Arsenite (trivalent, As[III]) is 5–10 times more toxic than arsenate (pentavalent, As[V]), which is thought to be mostly due to the higher solubility of arsenite (60). Importantly, arsenite can be transformed into arsenate in solutions and under specific conditions (61–63). We noticed that diluted arsenite (As[III]) stock solutions (e.g. 100 mM) lost potency over time both in regard to inducing cell death as well as tRNA fragmentation. In contrast, stock solutions containing high As[III] molarities (>1 M) induced robust cellular stress responses independently of storage time. Could previous interpretations regarding the effects of As[III] on stress responses, SG formation and tRNA fragmentation have been affected by the oxidative state of the used arsenic compounds? To test this, HeLa cells were exposed to 0.5 mM arsenic solutions containing different ratios of both forms (As[III] and As[V]) followed by determining SG formation, the induction of cell death as well as northern blotting for tRNA fragmentation. The results revealed that As[V] failed to induce SG at concentrations that were comparable to As[III] (Figure 7A). Furthermore, As[V] exposure barely resulted in tRNA fragmentation at concentrations at which As[III] induced robust tsRNA levels (Figure 7B). Notably, tRNA fragmentation could be induced by As[V] concentrations exceeding 1 mM (Figure 7C). Importantly, increased As[V] levels within the As[III]-As[V] mix abrogated the induction of cell death within 24 h after removal of arsenic insult (Figure 7D). These observations suggested that the outcome of experiments involving exposure of cells to arsenic compounds is depending on the oxidative state of the arsenic stock solutions.

## DISCUSSION

The biology of eukaryotic stress response pathways stretches across multiple disciplines from signaling to defense, from RNA biology to liquid-liquid phase transition, and from survival mechanisms to programmed cell death. Eukaryotic stress responses also include the phenomenon of stress-induced tRNA fragmentation. Even though cells employ various molecular machineries that detect and degrade aberrantly processed or modified tRNAs (reviewed in (64)), one seminal finding changed the view on tRNA-derived ‘degradation’ products. Specifically, it was reported that amino acid-starved *Tetrahymena* accumulated specific tRNA fragments that originated from hydrolysis in the anticodon loop (4). Ever since, the potential for biological function of stress-induced tRNA fragments is under intense scrutiny. Numerous reports have been published that associated stress-induced tsRNA abundance with biological impact. However, one particular aspect of modeling eukaryotic stress responses in cell culture has been unequivocally accepted without further consideration. This concerns the use of experimental paradigms, which involve high concentrations of specific chemicals causing oxidative stress. In particular, As[III] has regularly been used as the stressor of choice, not only to induce tRNA fragmentation but also to study the molecular details of eukaryotic stress responses including SG formation and concomitant translational changes (6,19,24,26,38,39,65–68).

Exposure to arsenic compounds is a well-known health concern and limiting the levels of arsenic-containing compounds in drinking water and food sources is the aim of many regulatory controls. Early studies on water from South African aquifers concluded that arsenic levels at concentrations of 100 μg/l (769.9 nM) or less did not produce ‘an undue burden’ on the human body (69). The WHO has issued guidelines recommending concentrations of arsenic compounds in drinking water that are 10-times lower (10 μg/l = 77 nM) (70). However, all academic reports on As[III]-induced tRNA fragmentation employed molar concentrations that were 4000 to 10 000-times higher than those deemed safe for potable water (Supplementary Table S1). Such discrepancies question how modeling stress responses (in general) and tsRNA biogenesis (in particular) by using As[III] actually reflects arsenic exposure *in situ*, especially when cells are embedded in complex tissues. Furthermore, the consequences of As[III] exposure, specifically in regard to SG formation, translational changes and tRNA fragmentation, have mostly been determined during the acute stress response (within the time window of actual As[III] treatment), thereby largely ignoring protracted effects on cell proliferation or viability after removal of the stressor.

The biological impact of stress-induced tsRNAs has largely been sought within the cells producing these small RNAs. For instance, particular tsRNAs (called tiRNAs) have been implicated in impairing translation initiation by binding to particular proteins likely through particular structural motifs such as G-quadruplexes (41,46,47). Furthermore, binding of tsRNAs to pro-apoptotic factors is thought to modulate apoptotic pathways during the acute stress response (71). The data presented here support the notion that transient exposure of various cell culture models to As[III], and other stressors resulting in oxidation, is detrimental to cellular metabolism and viability. Importantly, we report that the onset of tRNA fragmentation correlated with stress levels from which cells could not recover quantitatively, and appear to die as one consequence. This applies not only to As[III] exposure at molarities, which were deemed non-lethal (35) but also to H_2_O_2_ concentrations that were used in various studies including the seminal studies by (25,36,72). These findings also challenge the accepted and reiterated notion that stress-induced tsRNAs support cell survival, for instance, by blocking apoptosis (71) and therefore suggest that the production of endogenous tsRNAs is a rather late cellular response to lethal stress. Furthermore, we also report that tRNA fragmentation and SG formation are not co-current events, which suggests that tsRNAs are produced after cells responded to the As[III] insult through SG formation. Since SG formation in all tested cell lines occurred at As[III] concentrations that did not result in tRNA fragmentation during the acute stress response, these findings contradict another perpetuated notion about the function of tsRNAs in promoting SG assembly (40,45,46). Importantly, our attempts to address how many tsRNAs are required for the induction of SG formation, resulted in findings that challenge the current understanding as to how specific stress-induced tsRNAs impact the stress response. Notably, semi-quantitative determination of individual 5′ tsRNAs produced by time-limited As[III] exposure indicated that specific tsRNA species are detectable by northern blotting at copy numbers between 20 000 and 40 000 molecules per cell. Importantly, this value is likely an underestimation due to loss of RNA during extraction, blotting and probing. One can therefore assume that specific tsRNAs are present in cells at copy numbers that are comparable to those of specific miRNAs (73). However, these data also underscore the notion that the perceived impact of specific tsRNAs on cellular processes, must be mediated by those copy numbers that are inducible during the stress response and not by copy numbers, which often result from transfecting cells with non-physiological RNA mass. In this respect, it is curious that transfecting synthetic 5′ tsRNAs into cells resulted in copy numbers that exceeded 1 × 10^6^ molecules per cell, yet none of the transfected 5′ tsRNAs (alone or in combination, low to high mass) induced SG formation beyond background levels. Since physiological impact of transfected RNAs requires efficient release from endosomal compartments, we cannot exclude that the number of released and thereby physiologically relevant tsRNAs remained low, independently of the transfected RNA copy number. Furthermore, it has to be considered that synthetic tsRNA sequences might be a poor surrogate for endogenously produced tsRNAs, which contain various chemical modifications and could also be structured. Therefore, our data suggest that transfection of synthetic tsRNAs by lipofection is ill-suited for studying the effects of tsRNAs on SG formation or dissolution, but do not exclude that endogenously produced tsRNAs might be acting locally at specific subcellular sites to impact stress-related cellular physiology in a biologically meaningful fashion.

In addition, our observations point towards a disconnect between the low levels of specific 5′ tsRNAs in As[III]-stressed cells and their assumed role in suppressing protein synthesis in a general fashion (6,46). This is important since tRNA fragmentation (detectable by northern blotting) could only be elicited at As[III] concentrations above 0.2 mM while general protein synthesis inhibition has been reported to occur at As[III] concentrations below 0.1 mM (46,74), a concentration that also induces SG formation, especially in immortalized cells. Given the copy numbers of specific tsRNAs resulting from the limited endonucleolytic fragmentation of a given tRNA isoacceptor/isodecoder, one could assume that tRNA fragmentation during the acute stress response serves specific, likely localized, mechanistic purposes (*i.e*., inactivation of amino-acylated tRNAs, inhibiting/activating specific proteins with rate-limiting functions), which, nevertheless, are ‘masked’ by the deleterious effects caused by the massive oxidative damage in cells responding to the stress paradigm.

Notably, tsRNAs are detectable in extracellular space including serum, breast milk, semen and plant phloem (54,75–78) suggesting long half-lives and protection from RNases. Currently, it is assumed that stable extracellular tsRNAs are actively secreted from cells (58,77,79,80) rather than representing stable left-overs from dying cells. Recent findings indicated that cells do release full-length tRNAs even without the impact of stress exposure, which can become substrates of secreted activities such as RNase 1, resulting in the production of extracellular small RNAs including tsRNAs (ex-tsRNAs) that differ in size from stress-induced tsRNAs (81). Our findings confirm that medium collected from cells grown under steady-state conditions contain various ex-tsRNAs, which are slightly shorter than As[III]-induced tsRNAs. However, time-limited exposure to As[III] concentrations, ultimately resulting in increased yet delayed cell death, caused an increase in the accumulation of ex-tsRNAs in the medium when compared to steady-state levels. These findings indicated that As[III]-induced processes, specifically after the removal of the insult, cause the release of cellular material, including tRNAs and RNase 1, thereby resulting in the creation of ex-tsRNAs. Since the stability of ex-tsRNAs might be supported by particular RNA structures (30,79,82), these observations also suggest that the reported abundance of specific tsRNAs in various bodily fluids could be interpreted as the result of increased stress, cell turnover or cell death within that particular biological system under scrutiny.

**Figure 8.**
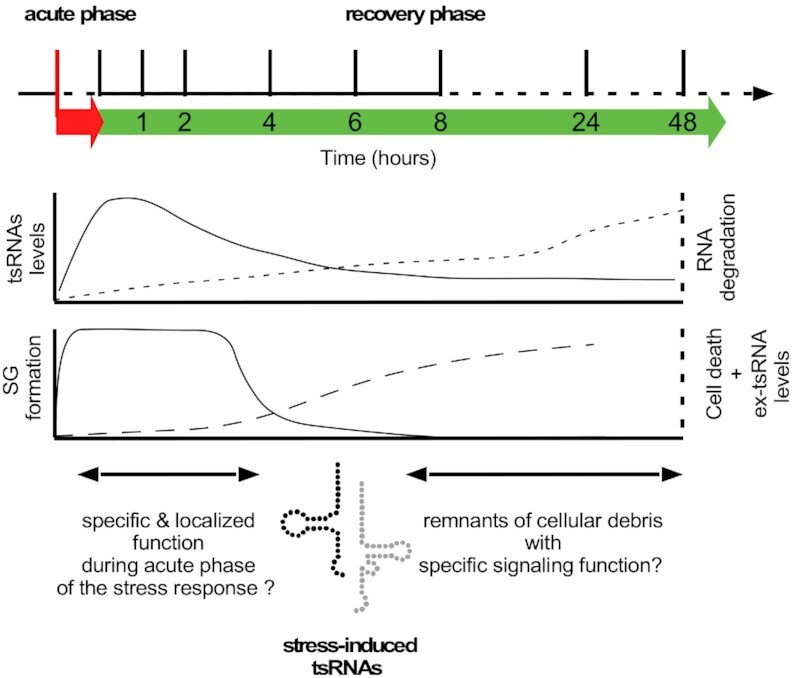
Cartoon depicting the general response of cultured cells to commonly used oxidative stressors such as As[III] or H_2_O_2_ during two major phases of the experimental manipulation. During the acute phase of the stress response, which, depending on the chemical stressor lasts from one to several hours (red arrow below dashed time line), cells respond with SG formation and an increase in tRNA fragmentation (lower graphs). Upon removal of the respective stressor and culture in recovery medium (green arrow), cells either survive or die, depending on the strength of the acute insult. While SG are dissolved within a few hours after stress removal, a large proportion of cells in recovery medium initiate pathways, which ultimately lead to loss of membrane integrity, increasing RNA degradation and cell death, which is concomitant with the appearance of stable ex-tsRNAs in the cell culture medium. During both phases, tsRNAs can be detected in cells using northern blotting. Since the peak of tsRNA signals is detectable during the acute phase, these small RNAs are likely exerting specific functions within cells related to acute stress responses. In contrast, the low level of tsRNAs that are produced after stress removal including the accumulation of ex-tsRNAs in cell culture media indicate that cell death-related processes contribute to the production of these small RNAs, which have either no biological function or play undefined roles as signaling molecules.

Taken together, our data suggest that when applying currently accepted stress paradigms for the induction of tRNA fragmentation, levels and functional impact of tsRNAs produced during the acute phase of the stress response have to be distinguished from those of tsRNAs and ex-tsRNAs, detectable after the removal of a respective stressor, since the latter might be the result of cellular repair processes, “waste management” or even cell death including further processing in extracellular space (Figure 8). We conclude that long-established stress paradigms applied to cell culture models allow studying the acute phase of the (oxidative) stress response including SG formation, protein translation changes, and, importantly the biogenesis of ANG-dependent as well as ANG-independent tsRNAs (26,34) but will likely fail revealing the impact of physiological levels of specific tsRNAs. Hence, our findings might guide future experiments that aim at elucidating the function and impact of stress-induced tsRNAs. To do so, it will be crucial to avoid stress levels that result in loss of cell viability. Furthermore, it might be prudent to also use animal stress models, which are often better suited for judging the severity of applied stress paradigms.

## SUPPLEMENTARY DATA

Supplementary Data are available at NAR Online.

## Supplementary Material

gkac495_Supplemental_Files
