# Supplementary material for: Experimental paradigms revisited: oxidative stress-induced tRNA fragmentation does not correlate with stress granule formation but is associated with delayed cell death
Source: Nucleic Acids Res. 2022 Jun 14;50(12):6919–37. doi: 10.1093/nar/gkac495 (PMC9262602; doi:10.1093/nar/gkac495)
Supplement: gkac495_Supplemental_Files [file gkac495_supplemental_files.zip › Supplementary_amended.pdf]

Sanadgol_Figure S1

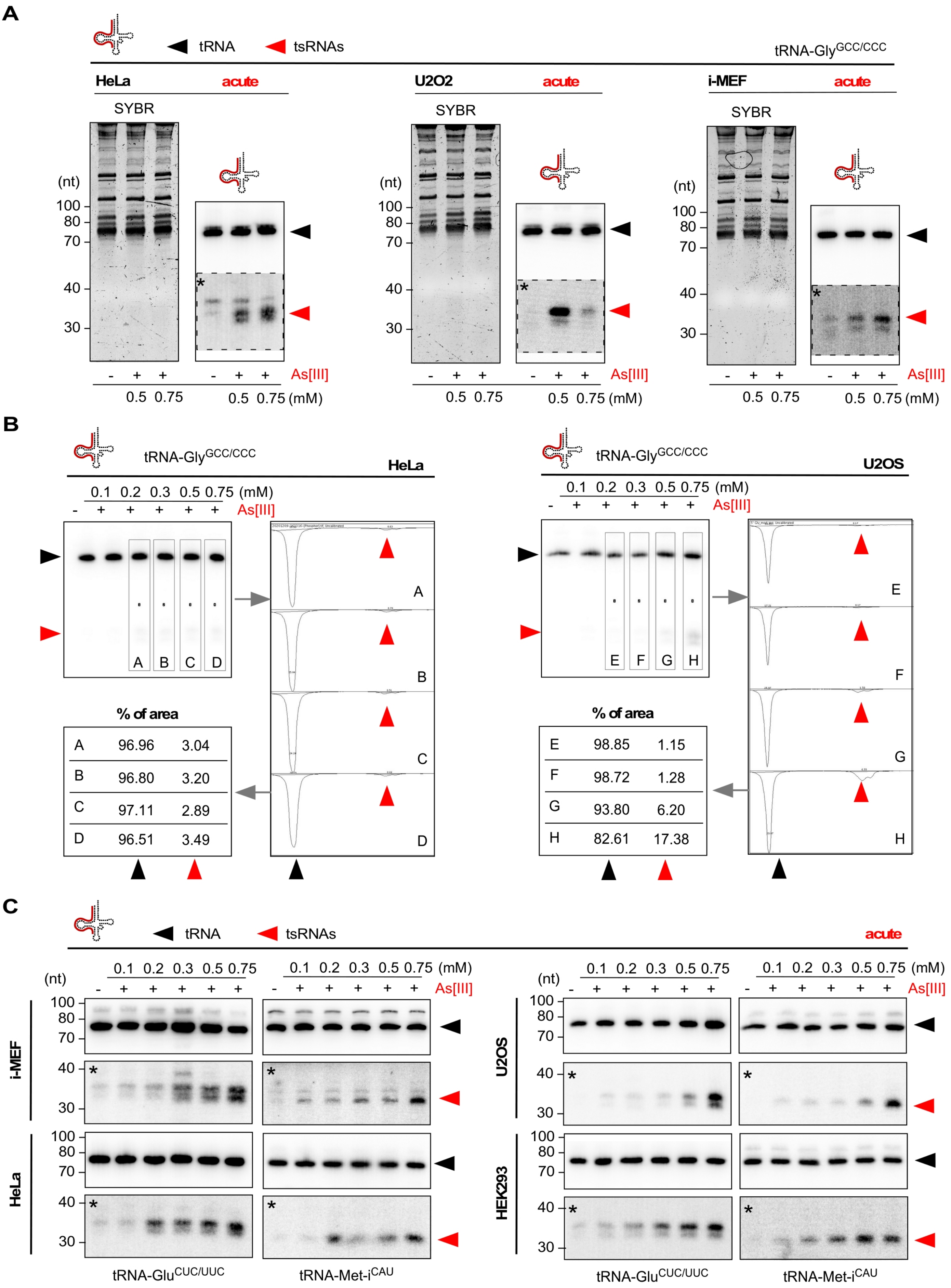

Supplementary Figure 1. As[III]-induced tRNA fragmentation results in cell type-dependent increase in tsRNA levels

(**A**) Northern blotting of total RNA (1.5 µg) from HeLa, U2OS cells and i-MEF exposed to ≥ 0.5 mM As[III] (for two hours, acute) using a probe against the 5' end of tRNA-$Gly^{GCC/CCC}$. Individual left images: SYBR-staining of PAA gels before transfer onto membranes. Black arrowheads: mature tRNAs; red arrowheads: 5' tsRNAs; asterisks: region that was digitally enhanced in lower panels.

(**B**) Relative quantification of specific As[III]-induced 5' tsRNAs through northern blotting of total RNA (1.5 µg) from HeLa cells (left) and U2OS cells (right) using a probe against the 5' end of tRNA-$Gly^{GCC/CCC}$ (related to **Figure 1A**). Images obtained by phospho-imaging were loaded into ImageJ without digital enhancement. Density distribution analysis of marked lanes (A-D and E-H, respectively) resulted in quantifiable peaks for tRNA signals (black arrowhead) and 5' tsRNAs (red arrowhead) representing the percentage of tRNA versus tsRNA.

(**C**) Northern blotting of total RNA (1.5 µg) from i-MEF, HeLa, U2OS, HEK293 cells that were exposed to increasing molarities of As[III] (0.1-0.75 mM, for one hour) using a probe against the 5' ends of tRNA-$Glu^{CUC/UUC}$ and tRNA-Met-i^{CAU}. Arrowheads and asterisks as in (A). Black arrowheads: mature tRNAs; red arrowheads: 5' tsRNAs; asterisks: region that was digitally enhanced in lower panels.

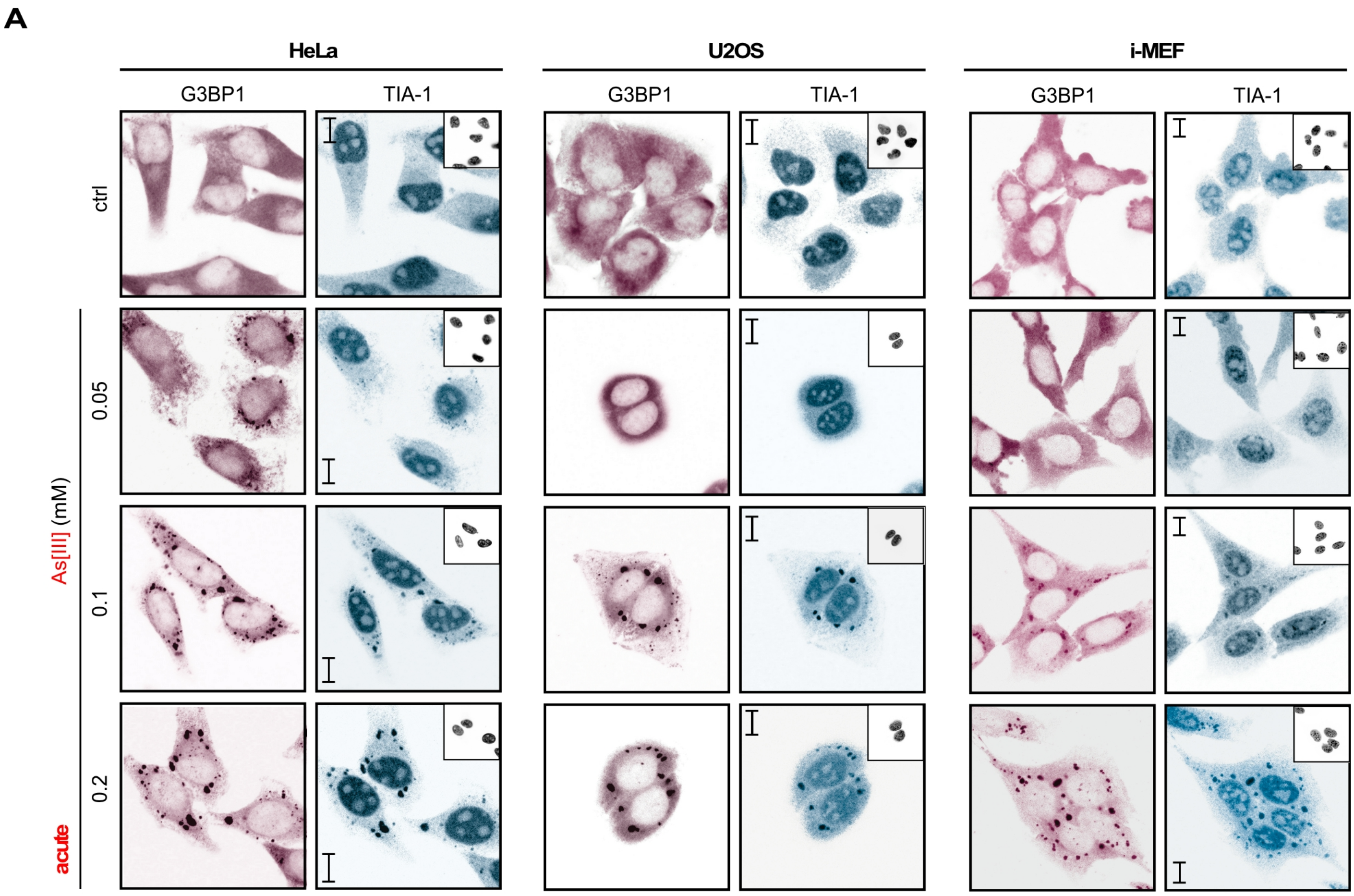
A
HeLa
U2OS
i-MEF
G3BP1
TIA-1
ctrl
0.05
0.1
0.2
As[III] (mM)
acute

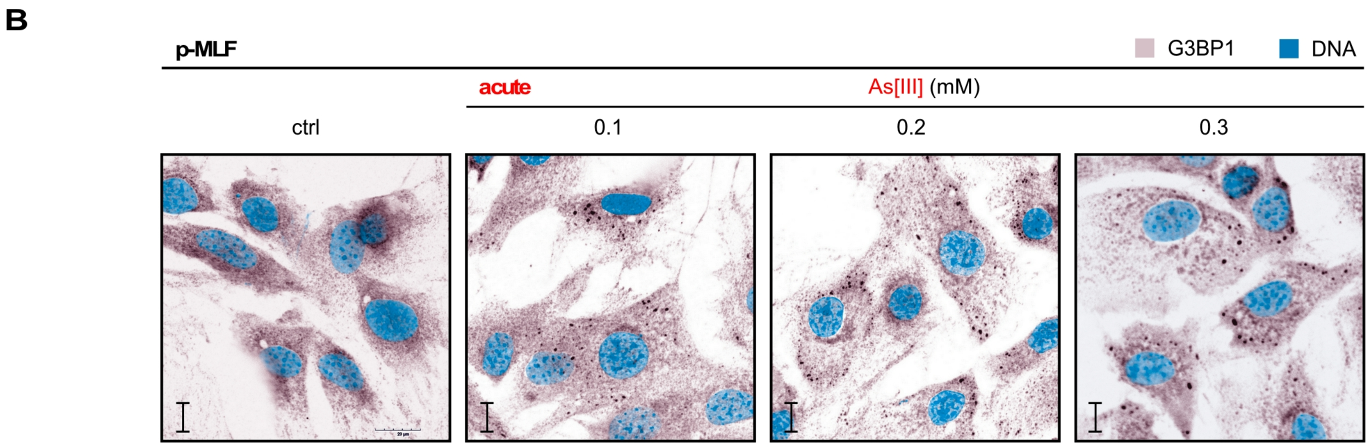
B
p-MLF
G3BP1
DNA
acute
As[III] (mM)
ctrl
0.1
0.2
0.3

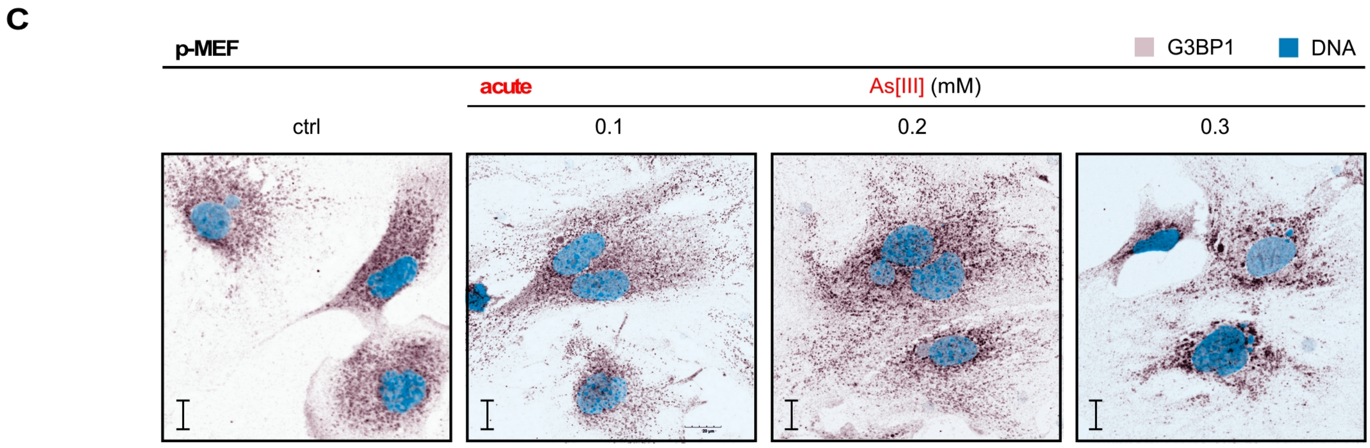
C
p-MEF
G3BP1
DNA
acute
As[III] (mM)
ctrl
0.1
0.2
0.3

Supplementary Figure 2. As[III]-induced stress granule formation differs between immortalized and primary cells

(**A**) Indirect immunofluorescence on HeLa, U2OS cells and i-MEF after time-limited exposure (one hour) to increasing As[III] molarities (0.05-0.2 mM) using antibodies against TIA-1 (cyan) and G3BP1 (magenta). Individual insets: DNA (black). Scale bar 10 μm.

(**B**) Indirect immunofluorescence on p-MLF after time-limited exposure (one hour) to increasing As[III] molarities (0.1-0.3 mM) using antibodies against G3BP1 (magenta). DNA is false-colored in cyan). Scale bar 10 μm.

(**C**) Indirect immunofluorescence on p-MEF after time-limited exposure (one hour) to increasing As[III] molarities (0.1-0.3 mM) using antibodies against G3BP1 (magenta). DNA is false-colored in cyan. Scale bar 10 μm.

Sanadgol_Figure S3

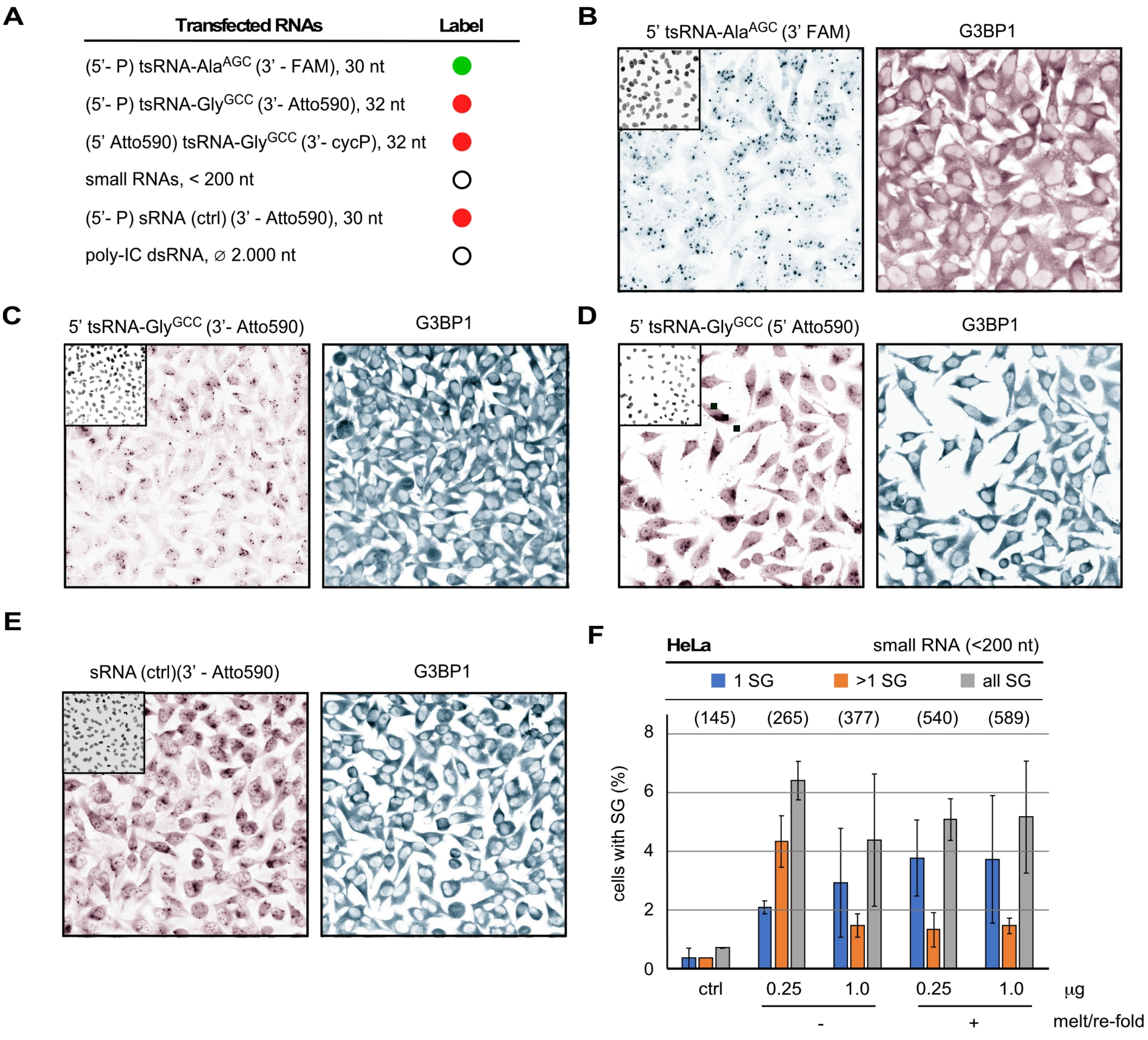

Supplementary Figure 3. 5' tsRNA transfection does not induce SG formation above RNA controls

(**A**) List of transfected RNAs that were used for images and analysis in **Figure 3** and **Supplementary Figure 3**.

(**B**) Representative confocal image of HeLa cells after transfection with 5' tsRNA-Ala^{AGC} labeled with FAM, 100 nM). Left image shows 5' tsRNA-Ala^{AGC} in cyan, while SG formation was monitored by indirect immunofluorescence using an antibody against G3BP1 (magenta). Inset: DNA (black).

(**C**) Representative confocal image of HeLa cells after transfection with 5' tsRNA-Gly^{GCC} labeled with Atto590 at the 3' end, 100 nM). Left image shows 5' tsRNA-Gly^{GCC} in magenta, while SG formation was monitored by indirect immunofluorescence using an antibody against G3BP1 (cyan). Inset: DNA (black).

(**D**) Representative confocal image of HeLa cells after transfection with 5' tsRNA-Gly^{GCC} labeled with Atto590, but containing a 3' CycP, 100 nM). Left image shows 5' tsRNA-Gly^{GCC} in magenta, while SG formation was monitored by indirect immunofluorescence using an antibody against G3BP1 (cyan). Inset: DNA (black).

(**E**) Representative confocal image of HeLa cells after transfection with small RNA as control (labeled with Atto590 at the 3' end, 100 nM). Left image shows small RNA signal in magenta, while SG formation was monitored by indirect immunofluorescence using an antibody against G3BP1 (cyan). Inset: DNA (black).

(**F**) Quantification of SG formed by transfection of HeLa cells with two masses (250 and 1000 ng) of small RNAs (< 200 nt) extracted from HeLa cells that were exposed for two hours to As[III] (0.5 mM), which were either denatured or melted and re-folded before transfection. Number of analyzed cells in parentheses.

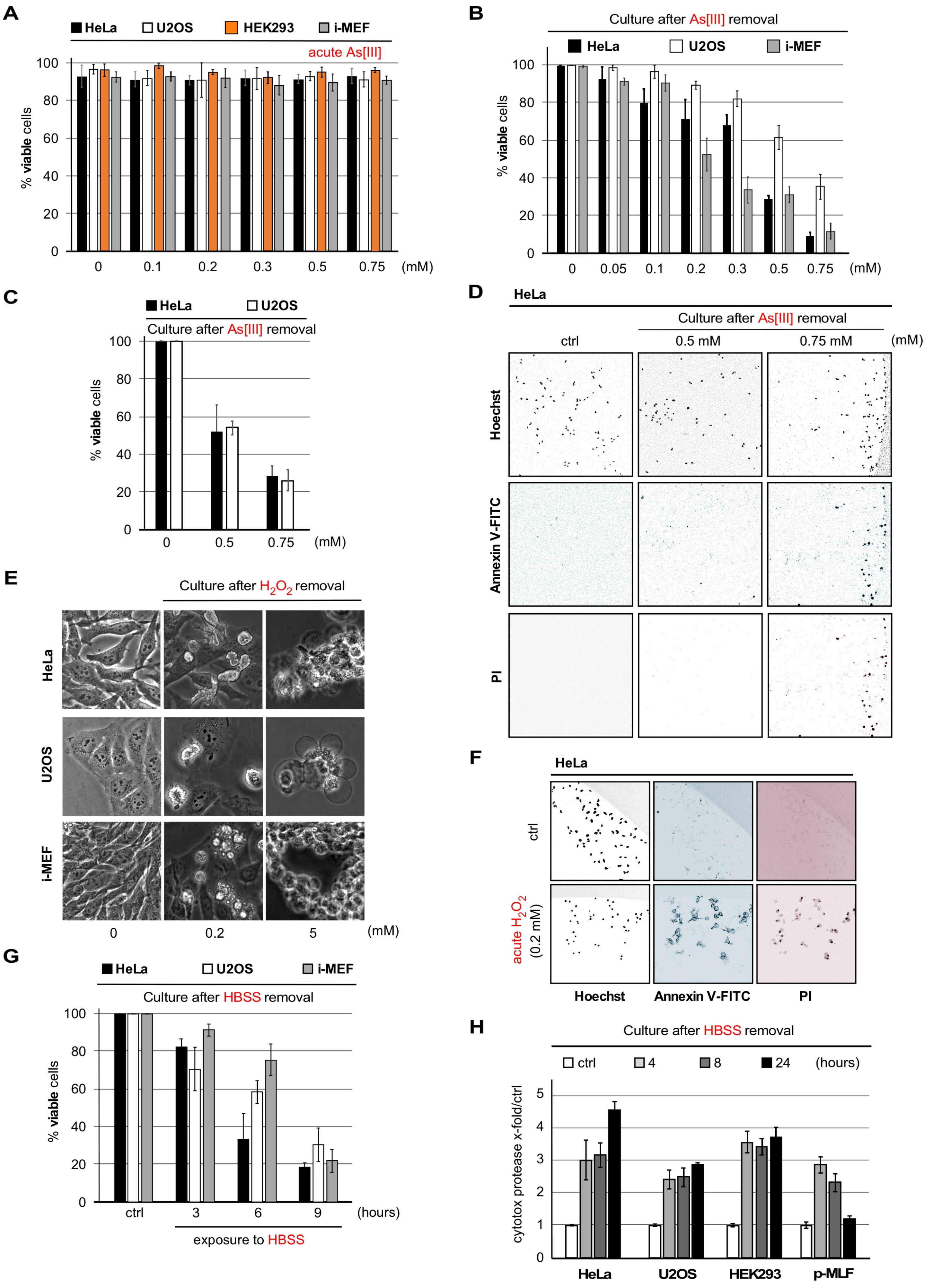
A
HeLa
U2OS
HEK293
i-MEF
acute As[III]
% viable cells
100
80
60
40
20
0
0.1
0.2
0.3
0.5
0.75
(mM)
B
Culture after As[III] removal
HeLa
U2OS
i-MEF
% viable cells
0.05
0.1
0.2
0.3
0.5
0.75
(mM)
C
HeLa
U2OS
Culture after As[III] removal
% viable cells
0
0.5
0.75
(mM)
D
HeLa
Culture after As[III] removal
ctrl
0.5 mM
0.75 mM
(mM)
Hoechst
Annexin V-FITC
PI
E
Culture after H2O2 removal
HeLa
U2OS
i-MEF
0
0.2
5
(mM)
F
HeLa
ctrl
acute H2O2
(0.2 mM)
Hoechst
Annexin V-FITC
PI
G
HeLa
U2OS
i-MEF
Culture after HBSS removal
% viable cells
ctrl
3
6
9
(hours)
exposure to HBSS
H
Culture after HBSS removal
ctrl
4
8
24
(hours)
cytotox protease x-fold/ctrl
5
4
3
2
1
0
HeLa
U2OS
HEK293
p-MLF

Supplementary Figure 4. Cell viability during the recovery from various stress paradigms

(**A**) Bar-chart depicting cell membrane-integrity measurements by Tblue staining of HeLa, U2OS, HEK293 cells and i-MEF immediately after time-limited exposure (one hour) to increasing molarities of As[III]. Error bars depict standard deviation (SD) of triplicate cell counts from three independent wells in the same experiment.

(**B**) Bar-chart depicting cell membrane-integrity measurements by Tblue staining of HeLa, U2OS cells and i-MEF after time-limited exposure (one hour) to increasing As[III] molarities, followed by culturing cells for 24 hours after the removal of As[III]. Error bars depict standard deviation (SD) of triplicate cell counts from three independent wells in the same experiment.

(**C**) Bar-chart depicting cell membrane-integrity measurements of HeLa and U2OS cells after exposure (two hours) to As[III] ≥ 0.5 mM as published in (6), followed by culturing cells for 24 hours after the removal of As[III]. Error bars depict standard deviation (SD) of triplicate cell counts from three independent wells in the same experiment.

(**D**) Representative images of living HeLa cells after exposure (two hours) to As[III] (0.5 and 0.75 mM) as published in (6), followed by culturing cells for 6 hours after the removal of As[III], and live-staining of cells using Annexin V-FITC, propidium iodide (PI) and Hoechst.

(**E**) Phase-contrast images of HeLa, U2OS cells and i-MEF after exposure (two hours) to H_2O_2 (0.2 or 5 mM), followed by culturing cells for 24 hours after the removal of H_2O_2 (related to **Figure 4F**).

(**F**) Representative images of living HeLa cells after exposure (two hours) to H_2O_2 (0.2 mM), followed by culturing cells for 6 hours after the removal of H_2O_2, and live-staining using Annexin V-FITC, propidium iodide (PI) and Hoechst.

(**G**) Bar-chart depicting cell membrane-integrity measurements by TBlue staining of HeLa, U2OS, HEK293 cells and i-MEF after being subjected to starvation through exposure to Hanks balanced salt solution (HBSS) for the indicated times (2, 6, 9 hours), followed by culturing cells for 24 hours after the removal of HBSS. Error bars depict standard deviation (SD) of triplicate cell counts from three independent wells in the same experiment.

(**H**) Bar-chart depicting the quantification of luminescence created by protease activity in the culture medium of HeLa, U2OS, HEK293 cells and p-MLF after exposure (3, 6, 9 hours) to HBSS, followed by culturing cells for 24 hours after the removal of HBSS. Error bars depict standard deviation (SD) of triplicate measurements from three independent wells in the same experiment.

Sanadgol_Figure S5

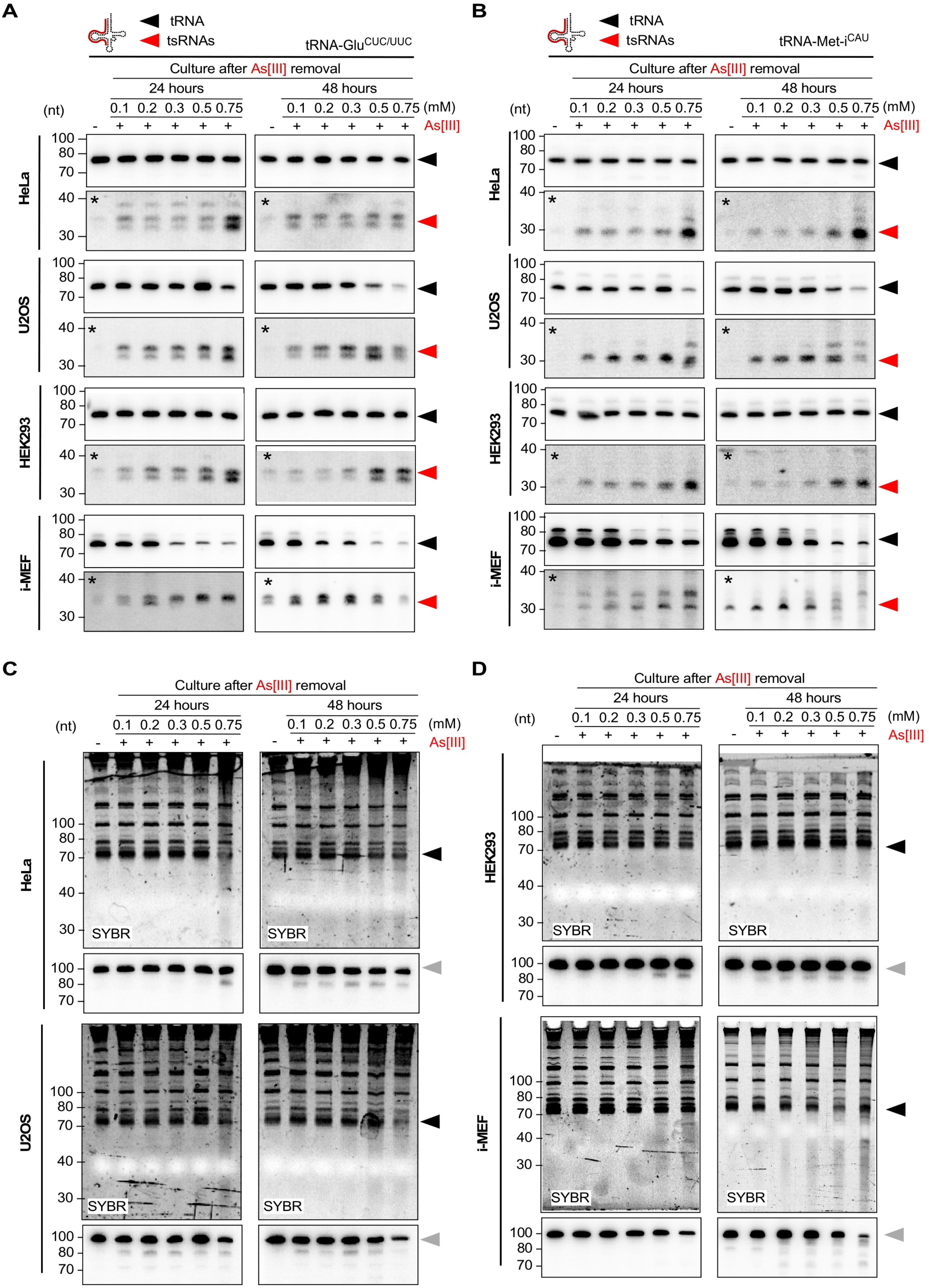

Supplementary Figure 5. tsRNA production and RNA integrity after removal of As[III]

(**A**) Northern blotting of total RNA (3 µg) extracted from HeLa, U2OS, HEK293 cells and i-MEF, which had been exposed (one hour) to increasing molarities of As[III], followed by culturing cells for 24 hours or 48 hours after the removal of As[III] using a probe against the 5' end of tRNA-Glu$^{CUC/UUC}$. Black arrowheads: mature tRNAs; red arrowheads: tsRNAs; asterisks: digitally enhanced against parental tRNA signals.

(**B**) Northern blotting of total RNA (3 µg) extracted from HeLa, U2OS, HEK293 cells and i-MEF, which had been exposed (one hour) to increasing molarities of As[III], followed by culturing cells for 24 hours or 48 hours after the removal of As[III] using a probe against the 5' end of tRNA-Met-i^{CAU}. Black arrowheads: mature tRNAs; red arrowheads: tsRNAs; asterisks: digitally enhanced against parental tRNA signals.

(**C**) SYBR-staining on PAA gels used for detecting tRNAs in HeLa and U2OS cells that were exposed to increasing molarities of As[III] (related to **Figure 5B** and **Supplementary Figure 5A, B**) and re-blotting of membranes for U6 snRNA Black arrowheads: mature tRNAs; grey arrowheads: U6 snRNA.

(**D**) SYBR-staining on PAA gels used for detecting tRNAs in HEK293 cells and i-MEF that were exposed to increasing molarities of As[III] (related to **Figure 5B** and **Supplementary Figure 5A, B**) and re-blotting of membranes for U6 snRNA Black arrowheads: mature tRNAs; grey arrowheads: U6 snRNA.

Sanadgol_Figure S6

A

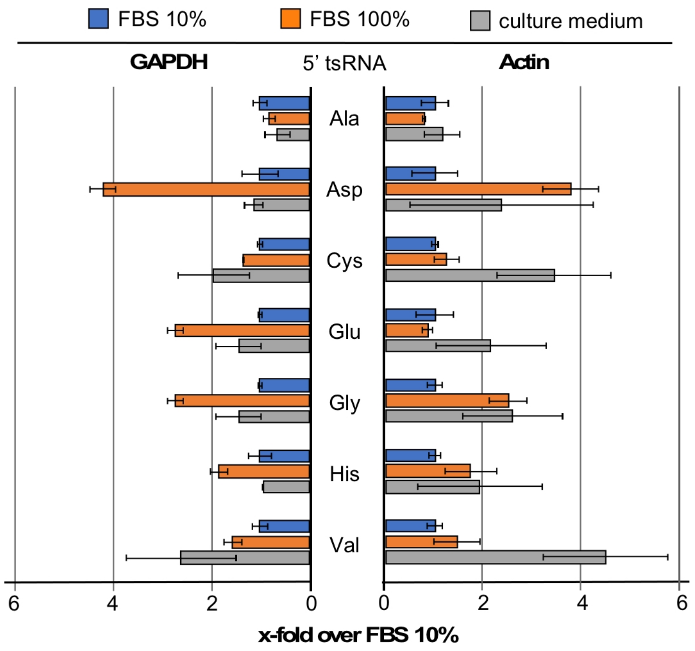

B

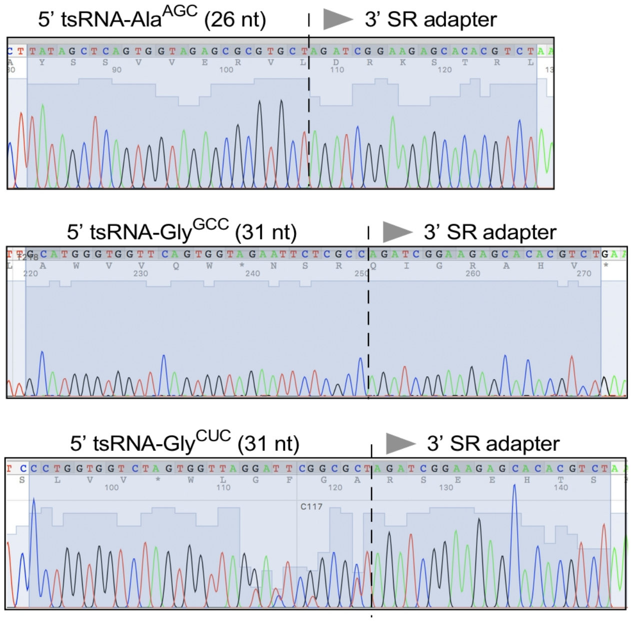

Supplementary Figure 6. Detection of 5' tsRNA in cell culture media

(**A**) qRT-PCR quantification of specific 5' tsRNAs contained in RNAs extracted from 40 µL FBS (equals the FBS percentage contained in 400 µL medium, 10%), 400 µL FBS, or 400 µL fresh cell culture medium. Bar-chart depicts the fold-change of specific 5' tsRNAs over FBS (10%) 40 µL FBS. Values were normalized to GAPDH (left) or ACTIN (right). Error bars depict standard error of the mean (SEM) of triplicate measurements from one experiment.

(**B**) Amplicons obtained from qRT-PCR on As[III]-conditioned HeLa cell culture medium were analyzed by Sanger-sequencing. Sequence traces reveal the size of individual extracellular tsRNAs. Vertical line: link to NEB 3' SR adapter revealing the 3' end of the respective tsRNAs

Supplementary Note 1

Emara, M. M.; Ivanov, P.; Hickman, T.; Dawra, N.; Tisdale, S.; Kedersha, N.; Hu, G.-F.; Anderson, P. Angiogenin-induced tRNA-derived stress-induced RNAs promote stress-induced stress granule assembly. ***J. Biol. Chem.*** **2010,** ***285*****, 10959–10968.**

From Material and Methods:

For tiRNA transfection, U2OS cells ($0.9*10^5$/well) were plated in 24-well plates for 24 h and then transfected with 750 nM synthetic or natural tiRNAs using 2.5 μL of Lipofectamine.

Calculation:

- $0.9*10^5$ cells seeded per 24 well plate/coverslip.
- Growth for one day -> $1.8*10^6$ cells
- Growth likely in medium volume of 0.5-1.0 mL per well
- transfected with 750 nM tiRNAs (natural or synthetic)

750 nM tiRNAs with average length between 30-40 nucleotides = 8.452 μg tiRNAs per mL

For 1 mL growth medium:

- 8.452 μg tiRNAs = $4.5*10^{14}$ tiRNA molecules
- $4.5*10^{14}$ tiRNA molecules in $1.8*10^6$ cells
- = **$2.5*10^8$ tiRNA molecules/cell**

For 0.5 mL growth medium:

- 4.226 μg tiRNAs = $2.25*10^{14}$ tiRNA molecules
- $2.25*10^{14}$ tiRNA molecules in $1.8*10^6$ cells
- = **$1.25*10^8$ tiRNA molecules/cell**

Yamasaki, S.; Ivanov, P.; Hu, G.-F.; Anderson, P. Angiogenin cleaves tRNA and promotes stress-induced translational repression. J Cell Biol 2009, 185, 35–42.

From Material and Methods:

In in vivo experiments, control RNA or tiRNAs were transfected into U2OS cells or MEFs in 24-well plates, and cultured for the indicated times. U2OS cells or MEFs were transfected with 5’ or 3’ tiRNAs (1 μM) using Lipofectamine 2000.

Calculation:

According to cell-culture-useful-numbers:

- $0.05*10^6$ cells seeded per 24 well plate/coverslip.
- Growth for one day -> $1.0*10^6$ cells
- Growth likely in medium volume of 0.5-1.0 mL per well
- transfected with 1 µM tiRNAs (natural)

1 µM tiRNAs with average length between 30-40 nucleotides = 11.27 µg tiRNAs per mL

For 1 mL growth medium:

- 11.27 µg tiRNAs = $6*10^{14}$ tiRNA molecules
- $6*10^{14}$ tiRNA molecules in $1.0*10^6$ cells
- = **$6*10^8$ tiRNA molecules/cell**

For 0.5 mL growth medium:

- 5.635 µg tiRNAs = $3*10^{14}$ tiRNA molecules
- $3*10^{14}$ tiRNA molecules in $1.0*10^6$ cells
- = **$3*10^8$ tiRNA molecules/cell**

Supplementary Table 1. List of reports using As[III] or H_2O_2 treatment for the induction of stress responses including tRNA fragmentation.

**Cell line**	**Chemical & Concentration**	**Time (hours)**	**Proof of tsRNAs**	**Recovery Analysis**	**Reference**
HeLa	H_2O_2 (5 mM)	4	yes	no	(1)
U2OS, HeLa, Cos7	As[III] (0.5 mM)	0.7-2	yes	yes	(2)
U2OS	As[III] (0.07 mM)	1	no	no	(3)
U2OS	As[III] (0.07 mM)	?	no	no	(4)
MEF	As[III] (0.5 mM)	2	?	no	(5)
HeLa	As[III] (0.1-0.5 mM)	1-4	yes	no	(6)
HeLa, LNCaP	As[III] (0.5 mM)	1	no	yes	(7)
Human dermal fibroblasts, Primary murine skin cells	As[III] (0.2 mM)	2-6	yes	no	(8)
HK-2	As[III] (0.5 mM)	1-4	yes	no	(9)
LKS, MyePro	As[III] (0.5 mM)	2	yes	no	(10)
PC12	As[III] (0.2-0.8 mM)<br>H_2O_2 (0.05-0.8 mM)	1-6<br>1-6	yes	no	(11)
HEK293T, HeLa	As[III] (0.5 mM)	2	yes	no	(12)
SH-SY5Y	As[III] (0.25 mM)	1	yes	no	(13)
A549, SAE	As[III] (0.001-0.01 mM)	6	yes	no	(14)
HEK293T,	As[III] (0.2-1 mM)	1	yes	no	(15)

U2OS					
Human dermal fibroblasts, Primary murine skin cells	As[III] (0.2 mM)	2-4	yes	no	(16)
Hepa 1-6, HeLa	As[III] (1 mM)	1	yes	no	(17)
HeLa	As[III] (0.1-0.5 mM)	6	yes	no	(18)
HAP1	As[III] (0.25 mM)	2	yes	no	(19)
HEK293	As[III] (0.5 mM)	1	yes	no	(20)
MCF-7, U2OS, DU145	As[III] (0.2-0.5 mM)	1	yes	no	(21)
U2OS, HeLa, Cos7	As[III] (0.5 mM)	1-4	yes	no	(22)
MCF10A	H_2O_2 (0.2 mM)	1-24	yes	no	(23)
B35	As[III] (0.4-1 mM)	4	yes	no	(24)
HeLa	As[III] (0.5 mM)	1	yes	yes	(25)
PC12, B35	As[III] (1 mM)	4-6	yes	no	(26)
U2OS	As[III] (0.5 mM)	2	yes	no	(27)

Supplementary References

1. Thompson,D.M., Lu,C., Green,P.J. and Parker,R. (2008) tRNA cleavage is a conserved response to oxidative stress in eukaryotes. *RNA*, **14**, 2095–2103.
2. Yamasaki,S., Ivanov,P., Hu,G.-F. and Anderson,P. (2009) Angiogenin cleaves tRNA and promotes stress-induced translational repression. *J Cell Biol*, **185**, 35–42.
3. Emara,M.M., Ivanov,P., Hickman,T., Dawra,N., Tisdale,S., Kedersha,N., Hu,G.-F. and Anderson,P. (2010) Angiogenin-induced tRNA-derived stress-induced RNAs promote stress-induced stress granule assembly. *J. Biol. Chem.*, **285**, 10959–10968.
4. Ivanov,P., Emara,M.M., Villen,J., Gygi,S.P. and Anderson,P. (2011) Angiogenin-induced tRNA fragments inhibit translation initiation. *Mol Cell*, **43**, 613–623.

5. Saikia,M., Krokowski,D., Guan,B.-J., Ivanov,P., Parisien,M., Hu,G.-F., Anderson,P., Pan,T. and Hatzoglou,M. (2012) Genome-wide identification and quantitative analysis of cleaved tRNA fragments induced by cellular stress. *Journal of Biological Chemistry*, **287**, 42708–42725.

6. Czech,A., Wende,S., Mörl,M., Pan,T. and Ignatova,Z. (2013) Reversible and rapid transfer-RNA deactivation as a mechanism of translational repression in stress. *PLoS Genet.*, **9**, e1003767.

7. Pizzo,E., Sarcinelli,C., Sheng,J., Fusco,S., Formiggini,F., Netti,P., Yu,W., D'Alessio,G. and Hu,G.-F. (2013) Ribonuclease/angiogenin inhibitor 1 regulates stress-induced subcellular localization of angiogenin to control growth and survival. *J. Cell. Sci.*, **126**, 4308–4319.

8. Blanco,S., Dietmann,S., Flores,J.V., Hussain,S., Kutter,C., Humphreys,P., Lukk,M., Lombard,P., Treps,L., Popis,M., *et al.* (2014) Aberrant methylation of tRNAs links cellular stress to neuro-developmental disorders. *EMBO J.*, **33**, 2020–2039.

9. Mishima,E., Jinno,D., Akiyama,Y., Itoh,K., Nankumo,S., Shima,H., Kikuchi,K., Takeuchi,Y., Elkordy,A., Suzuki,T., *et al.* (2015) Immuno-Northern Blotting: Detection of RNA Modifications by Using Antibodies against Modified Nucleosides. *PLoS ONE*, **10**, e0143756–.

10. Goncalves,K.A., Silberstein,L., Li,S., Severe,N., Hu,M.G., Yang,H., Scadden,D.T. and Hu,G.-F. (2016) Angiogenin Promotes Hematopoietic Regeneration by Dichotomously Regulating Quiescence of Stem and Progenitor Cells. *Cell*, **166**, 894–906.

11. Elkordy,A., Mishima,E., Niizuma,K., Akiyama,Y., Fujimura,M., Tominaga,T. and Abe,T. (2018) Stress-induced tRNA cleavage and tiRNA generation in rat neuronal PC12 cells. *J. Neurochem.*, **588**, 4297.

12. Wang,X., Matuszek,Z., Huang,Y., Parisien,M., Dai,Q., Clark,W., Schwartz,M.H. and Pan,T. (2018) Queuosine modification protects cognate tRNAs against ribonuclease cleavage. *RNA*, **24**, 1305–1313.

13. Li,S., Chen,Y., Sun,D., Bai,R., Gao,X., Yang,Y., Sheng,J. and Xu,Z. (2018) Angiogenin Prevents Progranulin A9D Mutation-Induced Neuronal-Like Cell Apoptosis Through Cleaving tRNAs into tiRNAs. *Mol. Neurobiol.*, **55**, 1338–1351.

14. Liu,S., Chen,Y., Ren,Y., Zhou,J., Ren,J., Lee,I. and Bao,X. (2018) A tRNA-derived RNA Fragment Plays an Important Role in the Mechanism of Arsenite -induced Cellular Responses. *Sci. Rep.*, **8**, 16838–9.

15. Su,Z., Kuscu,C., Malik,A., Shibata,E. and Dutta,A. (2019) Angiogenin generates specific stress-induced tRNA halves and is not involved in tRF-3-mediated gene silencing. *Journal of Biological Chemistry*, **294**, 16930–16941.

16. Gkatza,N.A., Castro,C., Harvey,R.F., Heiss,M., Popis,M.C., Blanco,S., Bornelöv,S., Sajini,A.A., Gleeson,J.G., Griffin,J.L., *et al.* (2019) Cytosine-5 RNA methylation links protein synthesis to cell metabolism. *PLoS Biol.*, **17**, e3000297.

17. Kim,H.K., Xu,J., Chu,K., Park,H., Jang,H., Li,P., Valdmanis,P.N., Zhang,Q.C. and Kay,M.A. (2019) A tRNA-Derived Small RNA Regulates Ribosomal Protein S28 Protein Levels after Translation Initiation in Humans and Mice. *CellReports*, **29**, 3816–3824.e4.

18. Chen,Z., Qi,M., Shen,B., Luo,G., Wu,Y., Li,J., Lu,Z., Zheng,Z., Dai,Q. and Wang,H. (2019) Transfer RNA demethylase ALKBH3 promotes cancer progression via induction of tRNA-derived small RNAs. *Nucleic Acids Res*, **47**, 2533–2545.

19. Vitali,P. and Kiss,T. (2019) Cooperative 2'-O-methylation of the wobble cytidine of human elongator tRNAMet(CAT) by a nucleolar and a Cajal body-specific box C/D RNP. *Genes Dev*, **33**, 741–746.

20. Drino,A., Oberbauer,V., Troger,C., Janisiw,E., Anrather,D., Hartl,M., Kaiser,S., Kellner,S. and Schaefer,M.R. (2020) Production and purification of endogenously modified tRNA-derived small RNAs. *RNA Biol*, **17**, 1104–1115.

21. Tosar,J.P., Segovia,M., Castellano,M., Gámbaro,F., Akiyama,Y., Fagúndez,P., Olivera,Á., Costa,B., Possi,T., Hill,M., *et al.* (2020) Fragmentation of extracellular

ribosomes and tRNAs shapes the extracellular RNAome. *Nucleic Acids Res*, **48**, 12874–12888.

22. Shigematsu,M. and Kirino,Y. (2020) Oxidative stress enhances the expression of 2‘,3’-cyclic phosphate-containing RNAs. *RNA Biol*, **17**, 1060–1069.
23. Huh,D., Passarelli,M.C., Gao,J., Dusmatova,S.N., Goin,C., Fish,L., Pinzaru,A.M., Molina,H., Ren,Z., McMillan,E.A., *et al.* (2021) A stress-induced tyrosine-tRNA depletion response mediates codon-based translational repression and growth suppression. *EMBO J.*, **40**, e106696.
24. Rashad,S., Han,X., Sato,K., Mishima,E., Abe,T., Tominaga,T. and Niizuma,K. (2020) The stress specific impact of ALKBH1 on tRNA cleavage and tiRNA generation. *RNA Biol*, **17**, 1092–1103.
25. Pereira,M., Ribeiro,D.R., Pinheiro,M.M., Ferreira,M., Kellner,S. and Soares,A.R. (2021) m5U54 tRNA Hypomodification by Lack of TRMT2A Drives the Generation of tRNA-Derived Small RNAs. *International Journal of Molecular Sciences*, **22**, 2941.
26. Rashad,S., Tominaga,T. and Niizuma,K. (2021) The cell and stress-specific canonical and noncanonical tRNA cleavage. *J Cell Physiol*, **236**, 3710–3724.
27. Akiyama,Y., Lyons,S.M., Fay,M.M., Tomioka,Y., Abe,T., Anderson,P.J. and Ivanov,P. (2022) Selective Cleavage at CCA Ends and Anticodon Loops of tRNAs by Stress-Induced RNases. *Front Mol Biosci*, **9**, 791094.
